# Multidimensional clinical evaluation of remimazolam versus propofol and dexmedetomidine: two systematic reviews and meta-analyses based on differentiated endpoints

**DOI:** 10.1186/s12871-026-03951-4

**Published:** 2026-05-28

**Authors:** Yangkun Li, Yuhao Zhang, Yong Wang

**Affiliations:** 1 Guangzhou University of Chinese Medicine, Guangzhou, China; 2 State Key Laboratory of Traditional Chinese Medicine Syndrome, Guangzhou, China; 3 Department of Anesthesiology, The First Affiliated Hospital of Guangzhou University of Chinese Medicine, Guangzhou, China

**Keywords:** Remimazolam, General anesthesia, Sedation, Safety, Delirium

## Abstract

**Background:**

Remimazolam is a novel ultra-short-acting benzodiazepine sedative, but its clinical positioning, delirium risk profile, and comparative efficacy against standard-of-care sedatives remain incompletely defined. This study aimed to comprehensively evaluate its efficacy and safety compared with propofol (Analysis A) and dexmedetomidine (Analysis B) across diverse clinical settings.

**Methods:**

Two independent meta-analyses were performed: Analysis A compared remimazolam vs. propofol in intubated surgical adults (primary endpoint: postoperative delirium incidence and recovery quality); Analysis B compared remimazolam vs. dexmedetomidine in perioperative/ICU adults with sedation (primary endpoint: time to achieve target sedation). Standard meta-analytic methods, trial sequential analysis (TSA), meta-regression, GRADE evidence grading, and heterogeneity source analysis were applied.

**Results:**

Analysis A (vs. Propofol): Remimazolam showed a delirium risk comparable to propofol (OR 1.06, 95% CI 0.78–1.45; Moderate certainty), a finding confirmed by TSA as robust. Quality of recovery (QoR-15) was similar between two agents (MD -1.85, 95% CI -7.01 to 3.31), though the certainty was very low due to very serious inconsistency and serious imprecision (I^2^ = 91.2%). Remimazolam significantly reduced the incidence of intraoperative hypotension.

Analysis B (vs. Dexmedetomidine): Remimazolam significantly hastened sedation onset (MD -4.78 min, 95%CI: -8.8 to -0.88). Regarding recovery, TSA confirmed firm evidence that remimazolam offers no clinically meaningful advantage (≥ 5 min) in time to full alertness. Incidences of PONV were comparable across all groups.

**Conclusions:**

Remimazolam is a safe and effective alternative to propofol for surgical anesthesia, offering superior hemodynamic stability without increasing the risk of postoperative delirium. Compared with dexmedetomidine, remimazolam provides a faster onset of sedation, an advantage that may be especially pronounced in patients with pre-existing agitation. It is well-suited for ambulatory, high-turnover surgery and hemodynamically vulnerable populations. Its long-term neurocognitive safety in ICU sedation requires further investigation.

**Supplementary Information:**

The online version contains supplementary material available at 10.1186/s12871-026-03951-4.

## Introduction

Modern anesthesia and intensive care medicine persistently require an ideal sedative, one characterized by rapid onset and offset, superior hemodynamic stability, minimal respiratory depression, and a favorable neurocognitive safety profile. For decades, propofol has remained the global standard for general anesthesia and procedural sedation due to its predictable pharmacokinetics and rapid recovery. However, propofol is frequently associated with dose-dependent cardiovascular depression and respiratory suppression, particularly in elderly or hemodynamically vulnerable populations [1].

Dexmedetomidine, a selective α2-adrenergic agonist, despite its prominence in procedural and ICU settings for providing “cooperative sedation” and opioid-sparing effects, its clinical utility is often hampered by a slow onset of action and the risk of significant bradycardia and hypotension [1].

Remimazolam, a novel ultra-short-acting benzodiazepine, undergoes rapid hydrolysis by non-specific tissue esterases (primarily carboxylesterase 1, CES1) into an inactive carboxylic acid metabolite [2]. This unique metabolic pathway theoretically ensures an organ-independent clearance, minimal risk of drug accumulation, and a predictable recovery profile regardless of the infusion duration. Besides, one advantage of remimazolam is that it can be reversed by flumazenil, which is unavailable with propofol or dexmedetomidine.

Despite the theoretically superior profile of remimazolam, its comparative clinical positioning against current standards of care remains incompletely defined, with conflicting evidence regarding its impact on high-order clinical endpoints. While individual trials have explored its efficacy, there is a lack of comprehensive evidence evaluating remimazolam through a multidimensional lens, specifically addressing its neurocognitive safety (postoperative delirium, POD) and recovery quality (QoR-15) compared to propofol, as well as its sedation efficiency (onset and offset speed) compared to dexmedetomidine. While previous meta-analyses have largely focused on statistical synthesis across broad datasets, there remains a need to define the clinical positioning of remimazolam through a multidimensional lens. Ranging from elective ambulatory surgery to high-acuity ICU mechanical ventilation, the performance of remimazolam varies based on patient-specific physiological stress and baseline conditions.

To address these knowledge gaps, we performed two independent yet complementary meta-analyses. Analysis A focuses on adult surgical patients requiring airway management (intubation/supraglottic devices), where remimazolam is compared against propofol as a primary anesthetic agent. The emphasis is placed on postoperative delirium and quality of recovery to determine if remimazolam can mitigate the neurocognitive burdens associated with traditional agent. Given the historical concerns regarding benzodiazepine-induced cognitive impairment, establishing this non-inferiority is essential for its adoption as a primary anesthetic. Analysis B compares remimazolam with dexmedetomidine across a spectrum of procedural and ICU sedation scenarios, focusing on the speed of achieving target sedation and the dynamics of recovery to demonstrate its operational superiority in sedation efficiency This study aims to provide a robust, evidence-based framework for the clinical positioning of remimazolam in personalized anesthesia practice.

## Methods

We confirmed the registration of our study protocol in the International Prospective Register of Systematic Reviews (PROSPERO) under the identification number CRD42025633255. The methodological approach followed the PRISMA (Preferred Reporting Items for Systematic Reviews and Meta-Analyses) guidelines [3].

### Search strategy

Relevant studies were identified through systematic searches of PubMed, Embase, Cochrane library, ClinicalTrials.gov, and Web of Science from inception to December 3rd, 2024 with no language restriction. The search strategy combined terms related to “remimazolam”, “propofol”, “dexmedetomidine”, “randomized controlled trial” using boolean operators. Two complete search strategies were provided in Supplementary Materials Appendix 1.

### Study selection & eligibility criteria

Two parallel, clinically complementary meta-analyses were pre-specified to address distinct clinical questions, with separate eligibility criteria defined for each analysis. Study screening and selection were performed independently by two reviewers in duplicate, with discrepancies resolved by consensus with the senior author. Full-text articles were retrieved for all studies that met the preliminary eligibility criteria, with final inclusion confirmed against the pre-specified criteria below:

1. Analysis A (Remimazolam vs. Propofol): Parallel-group randomized controlled trials (RCTs) published as full-text articles; Study population: Adult patients (≥ 18 years old) undergoing surgical anesthesia with airway management (tracheal intubation or supraglottic airway devices); Intervention: Remimazolam used for induction and maintenance of general anesthesia; Comparator: Propofol used for induction and maintenance of general anesthesia. Reported at least one of the pre-specified primary or secondary outcomes. Studies focusing solely on brief procedural sedation (e.g., gastrointestinal endoscopy) were excluded, as prior meta-analyses have extensively evaluated such settings.
  This analysis was designed to specifically evaluate remimazolam as a full general anesthetic agent in surgical patients requiring airway management, where propofol is the current global gold standard. Restricting to this setting ensures the results are directly relevant to routine general anesthesia practice.
2. Analysis B (Remimazolam vs. Dexmedetomidine): Included RCTs involving adult patients. Intervention with remimazolam and comparison with dexmedetomidine. No restrictions were placed on the clinical setting (encompassing both operating room and ICU environments) to ensure a comprehensive synthesis of the currently limited evidence base.

This analysis aimed to evaluate remimazolam as a sedative agent across the full spectrum of clinical sedation scenarios, where dexmedetomidine is the first-line agent. Given the limited number of RCTs directly comparing remimazolam and dexmedetomidine, broad inclusion was pre-specified to maximize the sample size and generalizability of the results.

### Outcomes and data extraction

Data extraction was independently performed by two reviewers using standardized Excel forms. For studies reporting median and range/interquartile range instead of mean and standard deviation, parameters were estimated using the method described by Wan et al. [4]. Continuous data reported in seconds were converted to minutes for consistency.

Extracted data included:

1. Study characteristics: First author, publication year, country, sample size, funding source (including pharmaceutical industry sponsorship), clinical setting, and surgical type.
2. Patient demographics: Age, body mass index (BMI), and American Society of Anesthesiologists (ASA) physical status classification.
3. Intervention details: Full dosage regimen (induction loading dose, maintenance infusion range) of remimazolam and comparator agents, use of flumazenil for remimazolam reversal.
4. Outcomes for Analysis A: Primary outcomes were Quality of Recovery (QoR-15) and the incidence of postoperative delirium. Secondary outcomes included hypotension, postoperative nausea and vomiting (PONV), and extubation time.
5. Outcomes for Analysis B: The primary outcome was the time to achieve adequate sedation. Secondary outcomes included time to full alertness, PONV, hypotension, and respiratory complications (e.g., desaturation, atelectasis, aspiration, or pneumonia if available).

The full extracted datasets for both analyses are available in Appendix 2 Table S3&S4.

### Statistical analysis

Data analysis was conducted using metafor package (4.8.0) [5] via R(4.5.2).For dichotomous outcomes, we used the odds ratio (OR) with 95% confidence intervals (CI) as the effect measure. For all continuous outcomes, we used the mean difference (MD) with 95% CI as the effect measure. Given the anticipated clinical and methodological heterogeneity across the included trials (e.g., variations in surgical types and dosing protocols), a random-effects model was applied for all pooled analyses. Heterogeneity was evaluated with the Cochran’s Q-test and I^2^ statistics [6]. Statistical significance was set at *P* < 0.05 [7].

Funnel plots were constructed to visually evaluate potential publication bias for Analysis A&B, and Egger’s test was performed when studies were available for an outcome (k ≥ 10) [8, 9].

### Sensitivity& subgroup analyses

Leave-one-out method sensitivity analyses were conducted for Analysis A&B to verify the outcome robustness and heterogeneity source.

Subgroup analyses were conducted as follows:

For Analysis A, based on procedure type to further elucidate potential clinical heterogeneity related to surgical or procedural characteristics if involved RCTs’ number > 2:

1. Breast Surgery (predominantly female patients)
2. Orthopedic Surgery
3. Neurosurgery
4. Head and Neck Surgery (including maxillofacial surgery, oral surgery, endoscopic sinus surgery, and open thyroidectomy)
5. Cardiac and Thoracic Surgery
6. Urologic Surgery
7. Laparoscopic Surgery
8. ICU

Additionally, for two outcomes of particular interest in Analysis A, further subgroup analyses were performed:

1. For postoperative delirium, studies were stratified by age (mean/median age ≥ 60 years), given the increased vulnerability of older patients to neurocognitive complications.
2. For extubation time, studies were stratified by routine use of flumazenil to evaluate the impact of pharmacological reversal on recovery.

For Analysis B, if insufficient data existed for pooled analysis by surgical type, subgroup analyses were performed according to clinical setting, including operating room, ICU, and endoscopic procedures.

### Trial sequential analysis & meta-regression

Trial Sequential Analysis (TSA) was performed to control the risk of type I and type II errors. We assumed an anticipated intervention effect of OR = 1.8 for binary outcomes and used the Minimal Clinically Important Difference (MCID) for continuous outcomes tailoring to each outcome: 8 points for QoR-15 [10], 5 min for extubation and full alertness times, and 4 min for sedation onset. These time-based thresholds were determined based on their potential impact on operating room turnover and procedural efficiency. The required information size (RIS) was calculated with an alpha of 0.05, a power of 90% [11], and adjusted for heterogeneity based on the I² statistic. The trial sequential monitoring boundaries were constructed using a Lan-DeMets alpha-spending function that mimics an O’Brien-Fleming sequential design [12].

For outcomes with extremely high heterogeneity (I^2^ > 75%) which cannot be resolved via sensitivity & subgroup analyses, random-effects meta-regression was performed to further investigate potential moderating effects (e.g., age, BMI, flumazenil used or not, flumazenil dosage reported by RCTs’ maximized dosage).

### Risk of bias and certainty of evidence

Risk of bias was evaluated using the Cochrane ROB 2 tool for all included RCTs. Assessments were performed independently by two reviewers, with disagreements resolved by a third reviewer.

We evaluated the certainty of the evidence for each outcome using the Grading of Recommendations, Assessment, Development, and Evaluation approach(GRADE [13]). Evidence certainty was categorized as high, moderate, low, or very low, based on five domains: risk of bias, inconsistency, indirectness, imprecision, and other considerations. As following:

- Inconsistency: If the I² statistic was > 50%, inconsistency was considered serious, and the evidence certainty was downgraded by one level. If I² >75%, inconsistency was considered very serious, and the certainty was downgraded by two levels.
- Indirectness: If there was substantial variation in the definition or measurement of an outcome across studies, indirectness was considered serious, and the certainty was downgraded by one level.
- Imprecision: Imprecision was considered serious if the 95% confidence interval of the pooled effect estimate was wide and crossed both the null effect and the pre-defined clinically important threshold for benefit or harm.
  For outcomes where the Trial Sequential Analysis (TSA) confirmed that the required information size (RIS) was met, imprecision was not downgraded, even if the 95% confidence interval crossed the null effect, as TSA indicated that the sample size was sufficient to rule out clinically meaningful differences.
- Publication bias: If publication bias was observed (e.g., *P* < 0.05 in Egger’s test or visual asymmetry in funnel plots), certainty was downgraded by one level.
- Risk of Bias: The certainty was downgraded by one level if more than 50% of the pooled weight was derived from studies rated as having “high risk” in the overall RoB 2 assessment. For studies with “some concerns”, we performed a qualitative assessment to determine if these concerns were likely to substantially bias the specific outcome; if not, the certainty was not downgraded to maintain the discriminatory power of the GRADE assessment in a clinical anesthesia context.

Regarding hypotension and respiratory complications, due to heterogeneous outcome definitions across included RCTs, we directly extracted and pooled the reported data as documented in each original study. Variability in diagnostic criteria was subsequently addressed during GRADE certainty assessment, with downgrading applied in the domain of indirectness to reflect clinical and methodological heterogeneity.

## Results

**Fig. 1:**
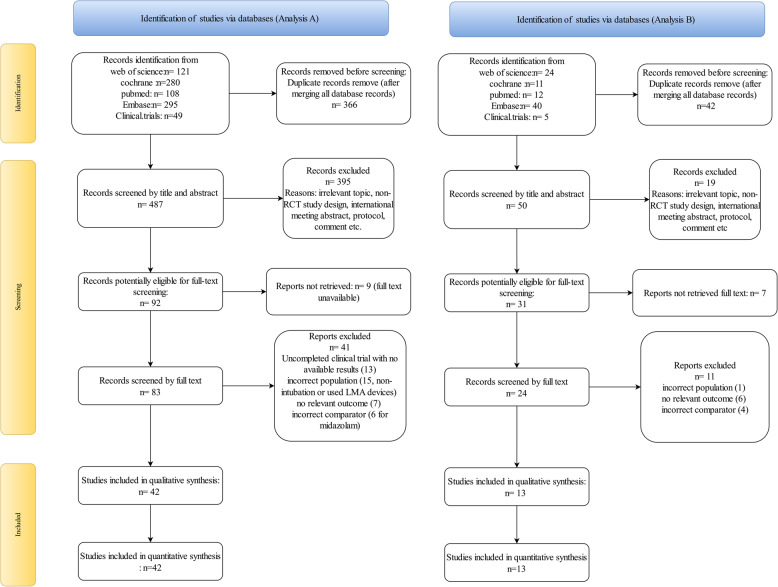
Selection processes of analysis A & B

Two study selection processes followed PRISMA guidelines and were illustrated in Fig. 1.

For Analysis A (Remimazolam vs. Propofol), the initial database search yielded 853 records. After removing 366 duplicates, 487 records were screened by title and abstract, of which 395 were excluded. Among the 92 potentially eligible reports, 9 were not retrieved due to full-text unavailability. The remaining 83 reports were assessed by full-text screening. 41 studies were further excluded as detailed in Fig. 1. Ultimately, 42 randomized controlled trials (RCTs) involving 5086 observations undergoing endotracheal intubation were included. Most studies came from Eastern Asian (China, Japan, South Korea), one from a multiple-nation RCTs (Germany, UK, Switzerland, Netherlands, Belgium, France, Austria). 29 of 42 declared funding support (including industry sponsorship), 13 did not. 1 was about ICU mechanical ventilation, the rest were conducted in the operating room for various elective surgical procedures (characteristics were detailed in Appendix 2-Table S1).

**Fig. 2:**
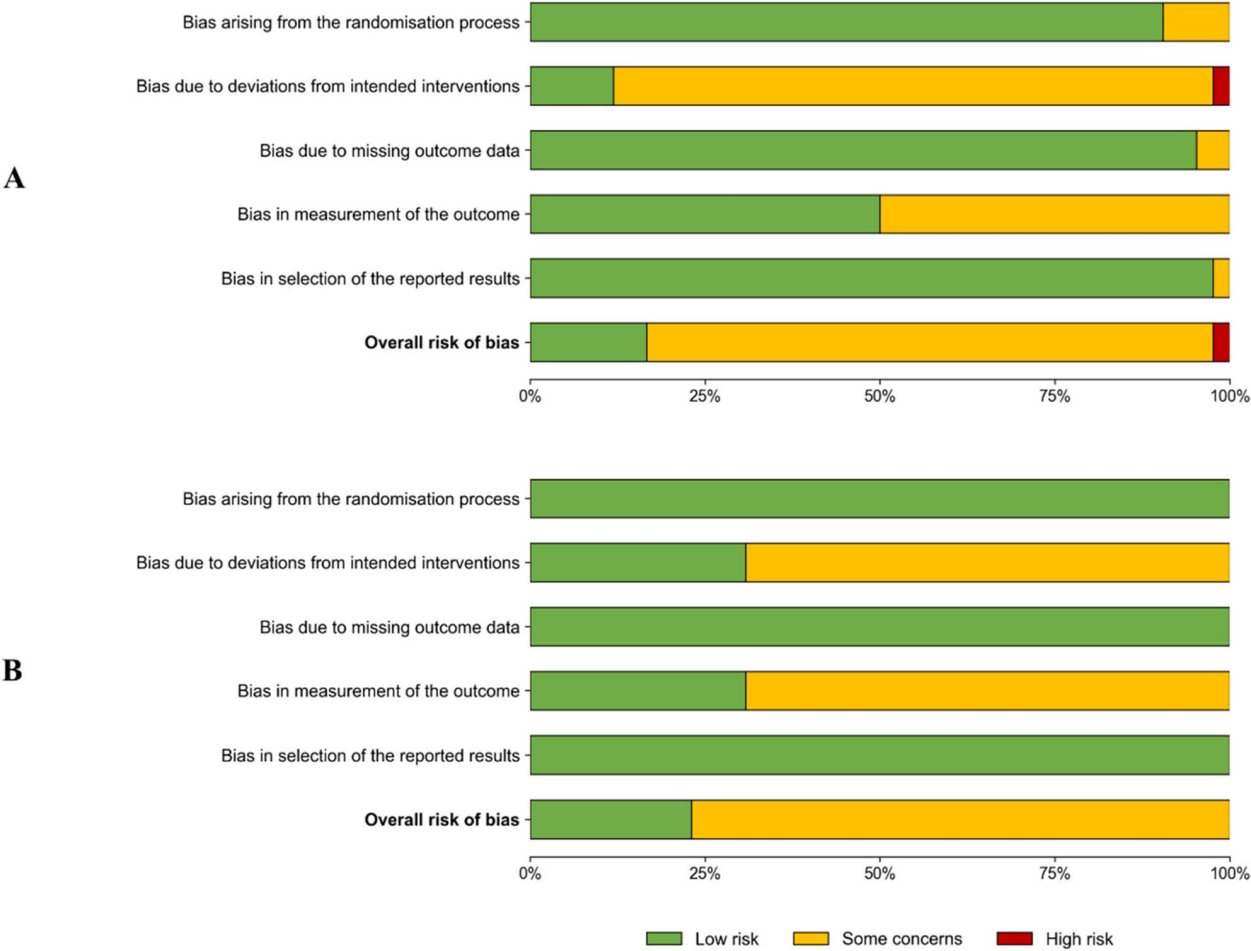
Risk of bias summary for Analysis A & B. **A** Risk of bias summary for Analysis A (Remimazolam vs. Propofol); (**B**) Risk of bias summary for Analysis B (Remimazolam vs. Dexmedetomidine). Details of every domain were shown in Appendix 3 Fig S1

The risk of bias across included studies of Analysis A was shown in Fig. 2A. Only approximately 20% of studies were rated as low risk of bias, while the majority (around 75%) raised some concerns. One study was rated as high risk of bias due to a lack of reporting on blinding of participants or personnel, additionally, the protocol allowed for rescue dosing that differed by group and violated the intended intervention without clarifying handling of protocol deviations. The overall RoB profile was primarily driven by concerns in the domains of deviations from intended interventions and outcome measurement (details of every domain were shown in Appendix 3 Fig S1).

For Analysis B (Remimazolam vs. Dexmedetomidine), 92 records were initially identified. After removing 42 duplicates, 50 records underwent title and abstract screening, resulting in 19 exclusions. Of the 31 reports sought for retrieval. 24 full-text articles were assessed for eligibility, and 11 were excluded as detailed in Fig. 1. Finally, total of 13 RCTs involving 1482 patients were included. 3 of 13 RCTs were conducted in patients undergoing bronchoscopy/endoscopy, 1 was mechanical ventilation in ICU setting, the rest were elective surgeries in operating room. 3 studies were not funded, while the rest were funded by county/council/nation projects. The mean age range was about 39–81. BMIs were within the normal range, with no trials exclusively recruiting underweight or obese populations (detailed in Appendix 2-Table S2).

Included studies of Analysis B were rated as low risk of bias in the domains of randomization process, missing outcome data, and selection of reported results (Fig. 2B). Due to some concerns about deviations from intended interventions (differential dosing regimens with insufficient reporting of blinding procedures), the majority (≈ 70%) raised some concerns. Furthermore, some studies did not mention blinding during assessing outcomes. Thus, approximately 25% of included RCTs were classified as low overall risk of overall bias, while 75% were rated as having some concerns.

### Analysis A: remimazolam vs. propofol

#### Primary outcome

##### Quality of Recovery (QoR-15)

**Fig. 3:**
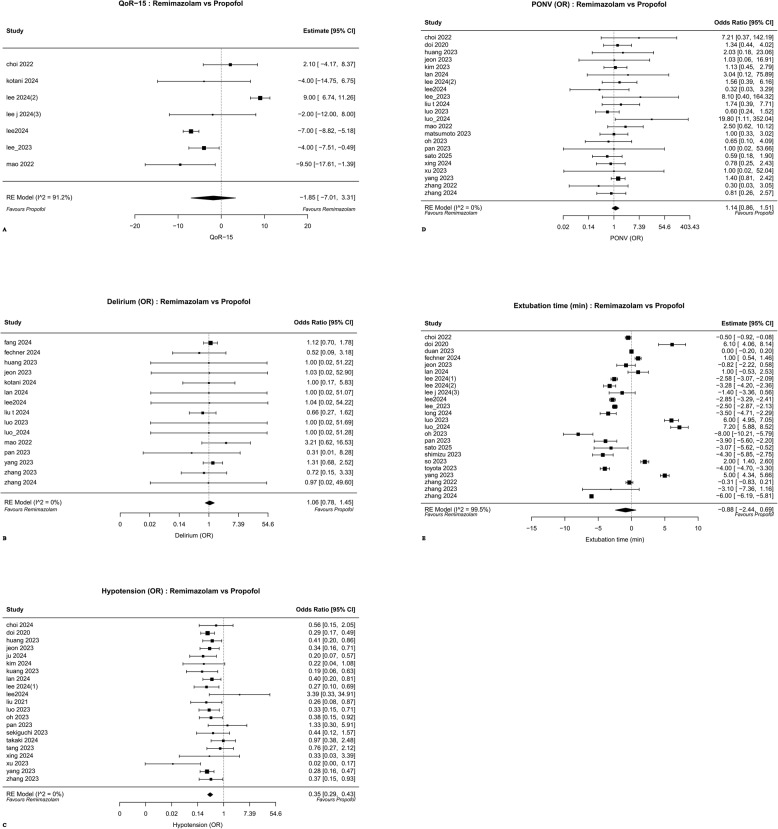
Forest plots for Analysis A (Remimazolam vs. Propofol). **A** Quality of Recovery-15 (QoR-15) score; (**B**) Delirium incidence; (**C**) Hypotension incidence; (**D**) Postoperative nausea and vomiting (PONV); (**E**) Extubation time (minutes). Continuous outcomes (**A**, **E**) are presented as mean differences (MD) with 95% confidence intervals. Dichotomous outcomes (**B**, **C**, **D**) are presented as odds ratios (OR) with 95% CIs. All analyses were performed using a random-effects model. For continuous outcomes, negative values favor remimazolam

Total 7 studies with 545 patients were over-pooled analyzed, forest plot in Fig. 3 showed a comparative QoR-15 score between Remimazolam and Propofol with high heterogeneity (I^2^ = 91.2%, MD -1.85, 95% CI [-7.01, 3.31]). Due to the small number of included studies (*n* = 7), formal tests for funnel plot asymmetry were not conducted, and no definitive conclusions regarding publication bias can be drawn from the visual inspection alone (Fig S2). Subgroup analyses based on funding status maintained consistency (Appendix 3, Fig S5).

A leave-one-out sensitivity analysis was conducted to investigate the high between-study heterogeneity, study of Lee et.al [14] was identified as the main source of heterogeneity; excluding it reduced the I² statistic (from 91.2% to 54.4%), whereas the overall pooled effect estimate showed statistically significant(MD -4.5 min, 95%CI -7.7 to -1.3).

Notably, this study enrolled only female patients undergoing breast cancer surgery, mainly Wide excision and Breast conservative surgery with procedures lasting 45 to 75 min (IQR). Specific gender and surgery type related hormonal changes may lead to a heterogeneity (detailed in discussion section).

**Fig. 4:**
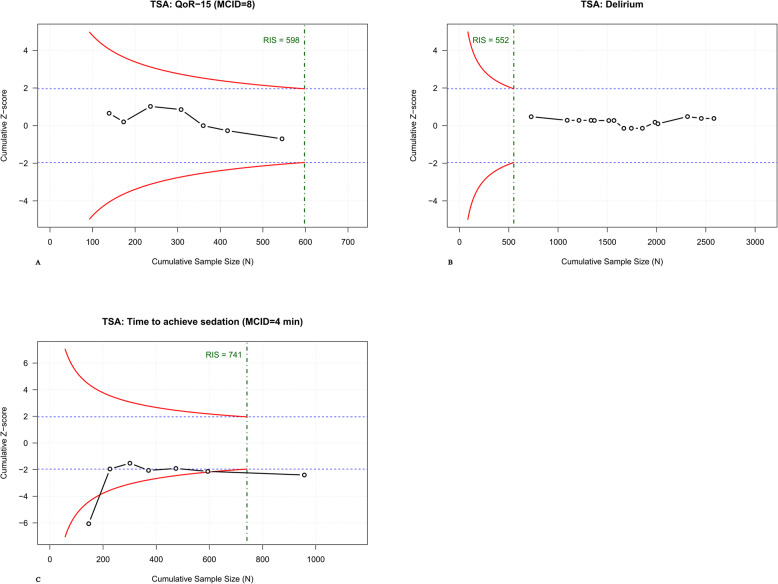
TSA outcomes of primary outcomes from Analysis A and B. **A** Quality of Recovery-15 (QoR-15) score (Analysis A), with a minimal clinically important difference (MCID) of 8 points; (**B**) Delirium incidence (Analysis A); (**C**) Time to achieve sedation (Analysis B), with an MCID of 4 min. The cumulative Z-curve (black line) was plotted against the required information size (RIS, green dashed line). The red curves represent the trial sequential monitoring boundaries for benefit or harm, and the blue horizontal lines indicate the conventional significance boundaries (two-sided a = 0.05)

According to previous literature [10], the Minimal Clinically Important Difference (MCID) was set to 8, and the Trial Sequential Analysis (TSA) outcome showed in Fig. 4A. The required information size (RIS) was calculated as 598 patients. The cumulative Z-curve (blue line) did not cross the traditional significance boundary (Z = 1.96) or the trial sequential monitoring boundary (red lines), nor did it reach the RIS. This indicates that current evidence is insufficient to reach a firm conclusion regarding a clinically meaningful difference between Remimazolam and Propofol. Thus, given all, the quality of evidence was rated as Very Low according to GRADE criteria (detailed in Table 1).

**Table 1:** Summary of findings (GRADE) for primary outcomes of analysis A & B

Outcomes	Studies, Patients	OR/MD (95% CI)	Certainty of evidence^#^	Reasons
Analysis A: Remimazolam vs. Propofol				
QoR-15 score	7, 545	-1.85; 95% CI (-7.01, 3.31)	⊕○○○ Very Low	Downgraded three levels: two for very serious inconsistency (I2 = 91.2%); one for serious imprecision (not reaching RIS), additional concerns regarding serious indirectness (procedural heterogeneity) were also noted, further undermining the reliability of this estimate.
Delirium	15, 2582	1.06; 95% CI (0.78, 1.45)	⊕⊕⊕○ Moderate	Downgraded one level for serious indirectness (diverse criteria across RCTs)
Analysis B: Remimazolam vs. Dexmedetomidine				
Time to Achieve Sedation (min)	7, 957	-4.78, 95%CI (-8.68, -0.88)	⊕○○○ Very Low	Downgraded three levels: two for very serious inconsistency (I^2^= 99.9%); one for serious indirectness (diverse criteria across RCTs)

*RIS* The required information size of Trial Sequential Analysis, *OR* odds ratio, *MD* mean difference, *95%CI* 95% confidence interval

^#^Certainty of evidence was judged according to GRADE approaches. Other GRADE results were shown in Appendix 2-Table S5

##### Delirium

Delirium was assessed in 15 RCTs (*N* = 2582) using Nu-DESC/CAM-ICU, with no significant difference between groups (OR: 1.06; 95% CI: 0.78 to 1.45; I^2^ = 0%). Funnel plot with Egger’s test showed no publication bias (*P* = 0.65, Fig S2-A). Leave-one-out method robustly confirmed this outcome during sensitivity analyses. Furthermore, subgroup analyses showed that no statistical significance was observed in research of mean/median age ≥ 60 populations (OR 1.1, 95%CI 0.63–1.94; 6 RCTs with 971 patients).

In TSA, cumulative sample size (*N* = 2582) significantly exceeded the RIS. The cumulative Z-curve remained well within the traditional and trial sequential monitoring boundaries, hovering near the zero line (Fig. 4B). These provide firm evidence that there is no clinically significant difference in the incidence of delirium between the Remimazolam and Propofol groups undergoing surgeries. The quality of evidence was rated as Moderate due to serious indirectness (diverse criteria across RCTs).

#### Secondary Outcomes

##### Hypotension

Meta-analysis of 21 studies (*N* = 2399) showed lower incidence of hypotension with Remimazolam compared to Propofol (OR: 0.35; 95% CI: 0.29 to 0.43; I^2^ = 0%). Sensitivity analyses and subgroup analyses confirmed the robustness of this result, and trial sequential analysis (TSA) further supported these findings (Fig S3-A). These results indicate that Remimazolam provides superior hemodynamic stability regardless of age. An odds ratio of 0.35 corresponds to a 65% relative reduction in the odds of hypotension with Remimazolam. No publication bias was observed (*P* = 0.54). Thus, the quality of evidence was Moderate due to serious indirectness (RCTs’ diverse criteria, Appendix 2-Table S5).

##### PONV

Analysis of 22 studies (*N* = 2632) showed no significant difference in postoperative nausea and vomiting (PONV) between Remimazolam and Propofol (OR 1.14; 95% CI 0.86 to 1.51; I² = 0%). No publication bias was observed (*P* = 0.44). TSA confirmed that the required information size (RIS) was reached, indicating that sufficient data were available to draw a firm conclusion. In surgical populations, regardless of age, the incidence of PONV was comparable between the two agents. The quality of evidence was rated as High according to the GRADE framework.

##### Extubation time

No significant difference was observed in terms of extubation time (minutes). 24 RCTs involving 2789 participants demonstrated comparable results between remimazolam and propofol (MD -0.88 min; 95% CI [-2.44, 0.69]). No publication bias was observed (*P* = 0.63). Heterogeneity was substantial (I² = 99.5%), and the source of this variance could not be identified through leave-one-out sensitivity analysis. Furthermore, subgroup analysis based on the administration of flumazenil (reversal agent) did not alter the non-significant findings, and heterogeneity remained substantial within both subgroups (both I^2^ > 90%). However, subgroup analyses by surgical type revealed substantial discrepancies across procedures (Appendix 3, Fig S6), suggesting that clinical heterogeneity among different surgeries may be the main source of instability.

According to GRADE framework, the evidence was rated as Very Low due to very serious inconsistency (I² = 99.5%) and serious indirectness related to variability in surgical procedures.

### Analysis B: Remimazolam vs. Dexmedetomidine

#### Primary outcome

##### Time to achieve sedation

**Fig. 5:**
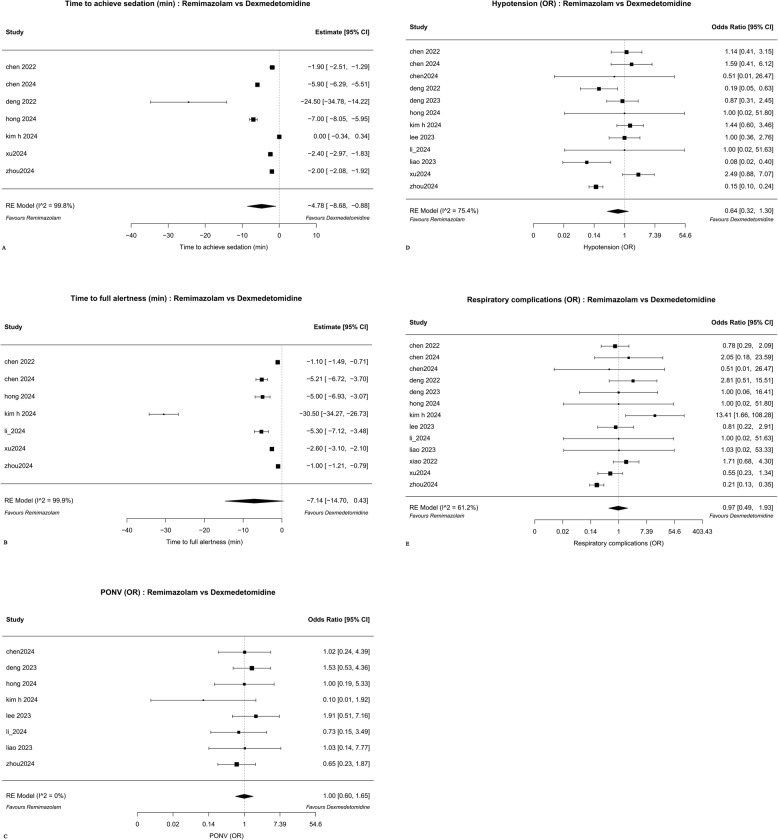
Forest plots for Analysis B. **A** Time to achieve sedation; (**B**) Time to full alertness; (**C**) PONV; (**D**) Hypotension incidence; (**E**) Desaturation. Continuous outcomes (**A**, **B**) are presented as mean differences (MD) with 95% confidence intervals. Dichotomous outcomes (**C**, **D**, **E**) are presented as odds ratios (OR) with 95% CIs. All analyses were performed using a random-effects model. For continuous outcomes, negative values favor remimazolam. (Remimazolam vs. Dexmedetomidine)

Remimazolam was associated with a significantly shorter onset of sedation compared to dexmedetomidine (MD: -4.78 min; 95% CI: -8.68 to -0.88; 7 RCTs; *n* = 957, Fig. 5A). Despite the uniform direction of effect favoring remimazolam across all trials, heterogeneity was remarkably high (I² = 99.9%). Although leave-one-out sensitivity analysis failed to identify a single source of the extreme heterogeneity, a clinical outlier was noted: Deng et al. [15] reported a substantially larger reduction in sedation onset (MD = -24.50 min; 95% CI: -34.78 to -14.22), potentially due to its specific ICU setting and the use of mechanical ventilation with pre-existing delirium or agitation populations. In contrast, other studies observed more modest reductions ranging from 2 to 7 min.

The TSA showed that the cumulative Z-curve crossed the traditional significance boundary and is currently hovering on the trial sequential monitoring boundary (Fig. 4C). Although the current sample size (*n* = 957) has surpassed the required information size (RIS = 741), firm evidence regarding a clinically meaningful advantage has not yet been fully established. This caution stems from the extreme heterogeneity and the sedation criteria differs among RCTs.

To explore the sources of heterogeneity, we performed single-covariate meta-regression using the Restricted Maximum Likelihood (REML) method. Neither mean age (R² = 0%, *P* = 0.12) nor mean BMI (R²=12.95%, *P* = 0.21) emerged as significant moderators, suggesting that these demographic factors did not account for the observed variance (Appendix-3-Fig S4).

Therefore, the results should be interpreted as suggestive rather than definitive, pending higher-quality, multi-center trials with more consistent protocols.

Due to the very serious inconsistency, serious indirectness, the quality of evidence for this outcome would likely be downgraded as Very Low according to GRADE criteria (Table 1).

#### Secondary outcomes

##### Time to full alertness

Regarding the time to full alertness, the pooled estimate from 7 studies (*n* = 962) showed no statistically significant difference between Remimazolam and Dexmedetomidine (MD: -7.14 min; 95% CI: -14.70 to 0.43, I^2^ = 99.9%). However, after excluding the study by Kim et al. [16], which reported a disproportionately large effect (MD = -30.50 min), the result became statistically significant (MD: -3.21 min; 95% CI: -4.86 to -1.56).

In the TSA (MCID = 5 min), although the required information size (RIS = 131) was exceeded, the cumulative Z-curve exhibited a telling trajectory: it initially crossed the monitoring boundary due to the influence of the outlier [16], but subsequently retreated into the non-significance zone as more trials were incorporated (Fig S3-B). Despite having adequate statistical power, firm evidence of a clinically meaningful advantage for remimazolam has not been established. This caution is warranted by the extreme heterogeneity, the fact that the adjusted MD (-3.21 min) remains below our pre-defined MCID, and the suspected publication bias suggested by the visual asymmetry in the funnel plot (Fig S2-B).

Given above, the evidence was graded as Very Low due to very serious heterogeneity and serious publication bias.

##### Postoperative Nausea and Vomiting (PONV)

Combined data from 8 studies (*n* = 960) showed no significant difference between groups (OR: 1; 95% CI: 0.6–1.65; I^2^ = 0%). The included trials spanned diverse clinical contexts, ranging from ICU settings to elective procedures such as orthopedic surgery and flexible fiberoptic bronchoscopy, involving both endotracheal intubation and procedural sedation. TSA further supported these findings, confirming that neither statistical nor clinical significance was observed. Given the consistency of the results across various settings and the adequate power, the certainty of evidence was graded as High.

##### Hypotension

12 studies involving 1381 patients reported the incidence of hypotension. The pooled estimate showed no statistically significant difference between remimazolam and dexmedetomidine (OR: 0.64; 95% CI: 0.32–1.30; I^2^= 75.4%). Sensitivity analysis was conducted due to high heterogeneity, finding that study by Zhou et al. [17] was a primary source of heterogeneity, though the pooled estimate remained non-significant upon its exclusion. TSA indicated that the cumulative sample size (*n* = 1381) had exceeded the RIS (*n* = 783), confirming that the evidence was sufficient to support a null finding. No publication bias was observed (*P* = 0.863). Considering the criteria of hypotension across RCTs differs, this evidence was graded as Very Low due to serious indirectness and very serious heterogeneity (I^2^ > 75%).

##### Respiratory complication - desaturation

12 studies involving 1401 patients reported the incidence of desaturation, which was the primary manifestation of respiratory complications in the included trials. Meta-analysis showed no significant difference between Remimazolam and Dexmedetomidine (OR: 0.98; 95% CI: 0.48 to 1.97, I^2^= 63.9%). Sensitivity analysis yielded consistent results, confirming the lack of statistical significance, and identified the study by Zhou et al. [17] was the main source of heterogeneity. TSA further confirmed the evidence was sufficient. No publication bias was observed (*P* = 0.15). The quality of evidence was Low due to serious inconsistency and indirectness (diverse criteria across RCTs).

Subgroup analyses of time-related outcomes based on settings were presented in Appendix 3 Fig S8.

## Discussion

This study comprises two comprehensive meta-analyses designed to evaluate the clinical profile of Remimazolam across diverse clinical settings and endpoints. Analysis A focused on the efficacy and safety of Remimazolam versus Propofol in adult patients requiring endotracheal intubation, with a primary emphasis on postoperative delirium and recovery quality. Analysis B compared remimazolam with dexmedetomidine in general adult populations across both intensive care and perioperative environments. By synthesizing evidence from these two distinct yet complementary perspectives, this study provides a relatively holistic assessment that may assist clinicians in optimizing sedative selection and improving patient outcomes.

### Interpretation of analysis A (vs. Propofol)

In surgical settings, remimazolam exhibited a delirium profile comparable to that of propofol (OR: 1.06; 95% CI: 0.78–1.45), supported by moderate-certainty evidence and confirmed by trial sequential analysis (TSA) as a robust null finding. This suggests that for perioperative sedation and anesthesia, remimazolam does not appear to carry the same delirium inducing burden like older benzodiazepines (e.g., midazolam). This favorable neurocognitive profile may be linked to its unique ester-based structure, which enables rapid, organ-independent metabolism via tissue esterases and minimizes drug accumulation during short-to-medium term administration.

Parallel to the delirium findings, the overall quality of recovery, as assessed by the QoR-15 scale, also showed no significant difference between remimazolam and propofol (MD: -1.85; 95% CI: -7.01 to 3.31). Unlike the high-certainty evidence for delirium, the certainty of evidence for QoR-15 was graded as very low, due to extreme between-study heterogeneity (I²=91.2%) and failure to reach the TSA-required information size (RIS). From a clinical standpoint, the pooled mean difference of -1.85 is substantially lower than the established minimal clinically important difference (MCID) of 8 points for the QoR-15 scale, indicating that remimazolam provides a similar recovery quality to propofol, but offers no superior clinical advantage in the immediate postoperative period.

Sensitivity analysis identified the study by Lee et al. [14] as the primary source of heterogeneity for the QoR-15 outcome. This trial exclusively enrolled female patients with breast cancer, a population in which pathological hormone alterations may induce changes in the expression of drug-related receptors and metabolic enzymes. We speculated that the alteration of GABA-A receptors and carboxylesterase 1 (CES1, the key enzyme responsible for remimazolam metabolism) in such a population [18–20], may alter drug sensitivity and lead to unintended drug accumulation, potentially explaining the divergent results in this cohort and the resulting heterogeneity in our pooled analysis.

Consistent with prior research, remimazolam was associated with a significantly lower incidence of intraoperative hypotension compared with propofol, representing a core safety advantage in clinical practice.

Notably, no significant difference in extubation time was observed between the two agents in our pooled analysis, irrespective of routine flumazenil administration. TSA utilized a pre-specified MCID of 5 min to define clinical relevance, confirmed that the current cumulative sample size has surpassed the required information size (RIS). This provides firm evidence that remimazolam offers no clinically meaningful advantage over propofol regarding extubation speed. Furthermore, the extreme heterogeneity associated with this outcome persisted across both the primary and subgroup analyses. This intractable variance is likely attributable to profound methodological heterogeneity in dosing protocols and surgery types: such as variations in opioid titration, neuromuscular blockade reversal strategies, and subjective institutional criteria for extubation readiness, rather than being solely driven by flumazenil use or dosage. This was further elucidated by our meta-regression analyses targeting flumazenil administration and maximum dosage, which failed to resolve the substantial residual heterogeneity (Appendix 3, Fig S7, R^2^= 9.13%, 5.62%. respectively). This indicates that extubation time is influenced by more complex, multifactorial determinants rather than by reversal agent use alone. Although subgroup analysis in neurological surgery suggested a potential benefit of remimazolam (MD: -2.84 min; Fig S6), this difference did not reach the pre-defined MCID of 5 min and thus lacked clinical significance.

Overall, for adult surgical patients, remimazolam is a favorable alternative to propofol in specific clinical scenarios, including ambulatory surgery, short-duration procedures, high-turnover surgical settings, and enhanced recovery after surgery (ERAS) pathways. Particularly in hemodynamically vulnerable patient populations, remimazolam is recommended given its superior hemodynamic stability and lower risk of perioperative hypotension.

Additionally, while our findings are robust for surgical anesthesia, evidence from the intensive care unit (ICU) setting, where long-term sedation (e.g., > 24–48 h) for mechanical ventilation, remains sparse. The majority of included trials in our meta-analysis focused on elective surgical procedures with a wide age range of patients (including those aged > 65 years), while data involving prolonged mechanical ventilation in the ICU were insufficient to draw definitive conclusions. Given that delirium risk is a complex outcome driven by the interaction of multiple predisposing and precipitating factors, and prolonged mechanical ventilation in the ICU is a well-established direct risk factor for delirium [21], the comparable delirium incidence observed in surgical cohorts cannot be reliably generalized to the intensive care setting.

Furthermore, although remimazolam is rapidly metabolized independently of the cytochrome P450 system [2, 22], the effects of prolonged administration (e.g., for continuous sedation in intubated ICU patients) on the activity of these metabolizing enzymes remain a subject of clinical debate. It is also unknown whether such long-term use may lead to drug accumulation, which could potentially contribute to delayed extubation or delayed awakening. Importantly, as a benzodiazepine agent, remimazolam belongs to a drug class recognized as an independent risk factor for delirium during prolonged ICU sedation. Therefore, its long-term safety in patients with predisposing risk factors for delirium has not been fully established. Beyond hemodynamics, safety evaluations should prioritize delirium as a critical indicator of long-term outcomes in vulnerable populations.

Future research specifically targeting long-term remimazolam infusions in critically ill patients is essential, to determine whether remimazolam maintains its favorable neurocognitive safety profile over extended administration periods.

### Interpretation of analysis B (vs. Dexmedetomidine)

In Analysis B, we compared remimazolam with dexmedetomidine, an α_2_-adrenergic agonist widely used for procedural and ICU sedation. Regarding sedation efficacy, remimazolam significantly reduced the time to achieve adequate sedation (MD: -4.78 min; 95% CI: -8.68 to -0.88). However, this finding was accompanied by extreme heterogeneity (I^2^ = 99.9%), which was likely driven by an ICU-based study (Deng et al. [15]) involving elderly patients with pre-existing delirium or agitation following orthopedic surgery. In this specific study, remimazolam achieved adequate sedation 24.5 min faster than dexmedetomidine (MD: -24.5 min, 95%CI: -34.78 to -14.22). On the contrary, in operating settings, the onset advantage of remimazolam was more modest (ranging from 2 to 7 min).

We speculated such a phenomenon may be fundamentally rooted in the distinct pharmacodynamic mechanisms of the two agents. Dexmedetomidine induces a state mimicking natural sleep via α2- adrenergic receptors in the locus coeruleus [1, 23]. In patients with hyperactive delirium, the profound sympathetic overdrive often overwhelms this mechanism [24–26]. Furthermore, the mandatory slow infusion of the dexmedetomidine loading dose (over 10–15 min to avoid hemodynamic collapse) inherently delays its onset. Conversely, remimazolam, as a GABA-A receptor agonist, provides direct and rapid central nervous system inhibition, effectively serving as an immediate restraint.

This profound mechanistic difference also explains why our initial meta-regression failed to identify mean age as a source of heterogeneity (R^2^ = 0%. Because Deng’s massive effect size was a product of the patients’ acutely agitated state rather than advanced age, age as a continuous variable lost its discriminatory power in the initial model (Appendix 3, Fig S4-B).

To validate this, we conducted a post-hoc sensitivity meta-regression excluding Deng et al., which suggested a potential age-dependent trend (R^2^ = 3.47%, *P* = 0.27), indicating that as patient age increases, the comparative advantage of remimazolam in onset time gradually narrows (shrinking from an MD of approximately − 7 min in younger cohorts to -2 minutes in older cohorts, Appendix3, Fig S4-C). Clinically, this aligns with the heightened sensitivity of the elderly brain to sedative agents. In younger, robust patients with high baseline sympathetic tone, the onset of dexmedetomidine is notably delayed, thereby maximizing remimazolam’s comparative speed advantage. In contrast, elderly patients are generally more susceptible to pharmacological suppression regardless of the agent used, naturally narrowing the pharmacokinetic gap between the two drugs. Given the small sample size and lack of statistical significance in the regression, these findings should be treated as exploratory hypotheses rather than definitive conclusions.

We also noticed the extremely high heterogeneity of time to full alertness (I^2^ = 99.9%), which likely originated from variations in dosage protocols and evaluation methods across studies. Specifically, in the outlier study by Kim et al. [16], the dexmedetomidine loading dose was 2-3fold higher than that in Chen et al. [27] (similar surgery with similar population characteristics), with total exposure varying by 1.5–2.5 fold (refer to Appendix 3-Table S2). This dosage protocol likely induced residual sedation, thereby inflating the recovery advantage of remimazolam. While remimazolam appears to facilitate faster alertness recovery, this advantage becomes less pronounced after excluding the outlier study. Our TSA, using a 5-minute MCID, further confirmed this observation; however, these findings warrant cautious interpretation due to the very low certainty of evidence.

While the TSA confirmed a lack of clinical significance based on the pre-specified OR threshold of 1.8, the hemodynamic advantages of remimazolam may be more pronounced in elderly and fragile populations with diminished sympathetic reserves. In these vulnerable patients, where maintaining cardiovascular stability is paramount, the superior safety profile of remimazolam may offer more meaningful clinical benefits than observed in younger cohorts.

Regarding the incidence of postoperative nausea and vomiting (PONV) and respiratory complications, current evidence suggests no significant differences between the two agents, indicating comparable safety profiles in these aspects.

### Summary of two analyses

Synthesizing the results of Analysis A and Analysis B reveals a comprehensive clinical profile for remimazolam. While Analysis A focuses on its role as a robust alternative to propofol in surgical anesthesia, Analysis B may highlight its advantages in sedation speed and cardiovascular safety over dexmedetomidine.

The traditional view in anesthesia is that benzodiazepines are independent risk factors for POD, particularly in the ICU. However, remimazolam’s ultra-short duration and organ-independent metabolism seem to bypass this classic disadvantage. Analysis A confirms a null delirium effect versus propofol, while external research suggests a potential benefit in frail patients, this result was confirmed by another similar meta-analysis by Xue et al. [28] in 2024 (RR,0.82; 95% CI 0.53–1.26; *P* = 0.36).

However, we compared these findings with a landmark meta-analysis by Zhou et al. [29] in 2025 included 17 RCTs with 3133 patients and found that remimazolam actually significantly decreased the risk of POD compared to propofol (OR: 0.71; 95% CI: 0.52–0.97, *P* = 0.03).This discrepancy with the null finding in Analysis A may be due to Zhou et al.’s inclusion of more recent trials focusing specifically on the elderly, where remimazolam’s hemodynamic stability provides the greatest neuroprotective advantage. Furthermore, Zhou et al. noted a trend toward greater benefit in studies with longer follow-up periods (e.g., 7 days), suggesting that remimazolam’s impact on cognition might be more evident during the later phases of recovery.

A major limitation identified in both Analysis A and B is the lack of robust data on long-term sedation (> 24–48 h) in the ICU. Most meta-analyses are dominated by elective surgical trials. However, the multicenter trial by Cheng et al. (2026) significantly addresses this gap [30]. In this study of 15 ICUs, remimazolam was non-inferior to dexmedetomidine in sedation efficacy for mechanically ventilated patients over 48 h (82.6% vs. 83.2%). Crucially, this multicenter trial confirmed the safety benefit seen in Analysis B: remimazolam patients had a much lower incidence of bradycardia (0.7% vs. 4.7%), which is related to hypotension as our result presented. This suggests that remimazolam is not only a viable procedural sedative but also a stable option for medium-to-long-term sedation in the ICU, particularly when dexmedetomidine-induced bradycardia or hypotension is a concern.

The high heterogeneity in Analysis A’s QoR-15 outcome (driven by Lee et al.) and Analysis B’s onset time (driven by Deng et al.) highlights the importance of patient-centered drug selection. The potential for altered metabolism in breast cancer patients, due to either pathological state or genetic polymorphisms in CES1, suggests that remimazolam’s ‘rapid recovery’ may not be universal. Research into Carboxylesterase 1 (CES1) genetic variants, such as the G143E polymorphism, has shown that some individuals may exhibit significantly impaired metabolism of remimazolam, leading to drug accumulation and prolonged sedation [31]. This provides a biological explanation for the ‘outlier’ results seen in certain trials and emphasizes the need for vigilant monitoring of sedation depth (e.g., BIS or RASS) rather than relying on standard weight-based dosing alone.

Another insight derived from this synthesis is that remimazolam’s value proposition may shift depending on the “physiological stress” of the patient. In Analysis A (Propofol), the primary advantage is safety (hemodynamics), whereas the outcome (delirium/recovery) is largely equivalent for the average patient. In Analysis B (Dexmedetomidine), the primary advantage is efficiency (onset speed). However, among the vulnerable patient in an acutely stressed state (e.g., the agitated elderly patient in the Deng et al. study), remimazolam may provide a unique combination of speed and safety that neither propofol nor dexmedetomidine can match. In these scenarios, the rapid CNS inhibition of a benzodiazepine is combined with the metabolic clearance of a soft drug, may minimize the time spent in a high-arousal, hyper-sympathetic state as we speculated.

### Limitations

Despite the comprehensive nature of this meta-analysis, several limitations must be acknowledged.

First, a high degree of heterogeneity was observed across multiple outcomes, particularly in sedation onset and recovery dynamics. Although we identified several clinical and statistical sources of this variance, such as dosing protocols, patient baseline agitation, and specific surgical populations (e.g., breast cancer), some residual heterogeneity remained unexplained, likely due to the inherent subjectivity in clinical sedation assessment scales.

Second, the number of studies conducted in the ICU setting for long-term sedation (> 24–48 h) remains limited. Consequently, our findings regarding neurocognitive safety (delirium) and drug accumulation are primarily derived from perioperative data and should be extrapolated to critically ill patients with caution.

Third, while we hypothesized a role for CES1 and GABA-A receptor alterations in specific cohorts, we lacked individual patient-level data to perform a more granular molecular-clinical correlation. Fourth, most included RCRs were conducted in East Asian populations, reflecting the current regional distribution of remimazolam-related clinical research. Thus, the generalizability of our findings to Western and non-East Asian cohorts is limited, and extrapolation to these populations should be done with caution.

Fifth, recovery endpoints like extubation and alertness times are susceptible to procedural confounders, such as surgical duration and site. Due to inconsistent reporting of anesthesia protocols (including neuromuscular blockade reversal and opioid titration), we could not fully adjust for these factors or resolve the associated heterogeneity. Consequently, these findings should be interpreted with caution.

Future research should prioritize large-scale, multicenter RCTs focusing on long-term remimazolam infusions in the ICU, with a specific emphasis on long-term neurocognitive trajectories and survival outcomes (e.g., 30–90 days cognitive status). Additionally, studies investigating the pharmacogenomics of remimazolam, specifically the impact of CES1 polymorphisms on metabolism, could help clinicians move toward a more personalized approach to sedation.

## Conclusion

Our findings demonstrate that remimazolam is a safe and effective alternative to propofol for surgical anesthesia, offering superior hemodynamic stability without increasing the risk of postoperative delirium. Compared with dexmedetomidine, remimazolam provides a faster onset of sedation, may particularly in patients with pre-existing agitation.

## Supplementary Information

Supplementary Material 1: Appendix 1: Search strategies for Analysis A&B. Appendix 2: All tables (including extracted datasets, characteristics of studies with references). Appendix 3: All supplementary figures (Fig S1-S8). Appendix 4: PRISMA checklist.

## Data Availability

This study is a systematic review and meta-analysis based on previously published randomized controlled trials. All primary data used in this study are available in the original published articles cited in the reference list. The extracted study-level datasets in this study are available in the Supplementary Materials of this manuscript.
