# Supplementary material for: Multidimensional clinical evaluation of remimazolam versus propofol and dexmedetomidine: two systematic reviews and meta-analyses based on differentiated endpoints
Source: BMC Anesthesiol. 2026 May 28;26:447. doi: 10.1186/s12871-026-03951-4 (PMC13397694; doi:10.1186/s12871-026-03951-4)
Supplement: Supplementary file 1 — Supplementary Material 1: Appendix 1: Search strategies for Analysis A&B. Appendix 2: All tables (including extracted datasets, characteristics of studies with references). Appendix 3: All supplementary figures (Fig S1-S8). Appendix 4: PRISMA checklist. [file 12871_2026_3951_MOESM1_ESM.zip › Appendix-2/Table_S1-S2.docx]

**Table S1. Characteristics of Analysis A included studies (42 RCTs)**

| **study_id** | **country** | **clinical_setting** | **surgery/operation** | **funding_status** | **remi_n** | **remi_dose** | **remi_age (mean/median)** | **remi_bmi (mean/median)** | **remi_asa_status** | **propofol_n** | **propofol_dose** | **propofol_age (mean/median)** | **propofol_bmi (mean/median)** | **propofol_asa_status** |
| --- | --- | --- | --- | --- | --- | --- | --- | --- | --- | --- | --- | --- | --- | --- |
| Choi 2022[1] | South Korea | operating room | open thyroidectomy | No | 70 | Induction 6 mg/kg/h, Maintenance 1-2 mg/kg/h | 39.5 |  | ASA I/II/III: 51/19/0 | 69 | Induction Ce 5 μg/mL, Maintenance Ce 2-6 μg/mL | 41 |  | ASA I/II/III: 50/19/0 |
| Choi 2024[2] | South Korea | operating room | Neurosurgical procedures (e.g., craniotomy, microvascular decompression, brain biopsy) | Yes(pharmaceutics: Hana Pharmaceuticals, Seoul, South Korea) | 48 | Induction: 6 mg/kg/h (flow rate), Maintenance: titrated to target PSI 40 | 60.9 |  | ASA II/III: 28/20 | 46 | Induction: TCI Marsh model, effect-site concentration 4 mcg/ml, Maintenance: titrated to target PSI 40 | 64.3 |  | ASA II/III: 19/27 |
| Doi 2020[3] | Japan | operating room | hospital stay ≥3 days, including extremities and lower abdomen surgery | Yes(pharmaceutics: Ono Pharmaceutical Co.) | 300 | Induction: 6 mg/kg/h -12 mg/kg/h ; Maintenance: 1 mg/kg/h (max 2 mg/kg/h) | 56.95 | 23.25 | ASA I/II: 146/154 | 75 | Induction: 2.0-2.5 mg/kg; Maintenance: 4-10 mg/kg/h | 56.3 | 23.3 | ASA I/II: 37/38 |
| Duan 2023[4] | China | operating room | Hip replacement surgery | No | 30 | Induction 0.2-0.4 mg/kg, Maintenance 0.3-0.5 mg/kg/h | 67.8 | 22.9 | ASA I/II/III: 4/20/6 | 30 | Induction 1.5-2 mg/kg, Maintenance 4-8 mg/kg/h | 68.7 | 23.3 | ASA I/II/III: 3/23/4 |
| Fang 2024[5] | China | operating room | Hip surgery | Yes(country/nation non-commercial) | 364 | Induction 0.2 to 0.25 mg/kg, Maintenance continuous intravenous infusion titrated to BIS 45-60 | | |  | 364 | Induction 1.5 to 2.0 mg/kg, Maintenance continuous intravenous infusion titrated to BIS 45-60 | | |  |
| Fechner 2024[6] | Multicentre (7 European countries: Germany, UK, Switzerland, Netherlands, Belgium, France, Austria) | operating room | Non-cardiac surgery (gastrointestinal, vascular, and urological procedures) | Yes(pharmaceutics: PAION Ltd., Cambridge, UK) | 270 | Induction: 6.0 mg.min-1 (0-3 min), 2.5 mg.min-1 (3-10 min), 1.5 mg.min-1 (10-20 min), 1.0 mg.min-1 (20 min onwards); Maintenance: 0.7-2.5 mg.min-1 (from 15 min after skin incision until end of surgery); | 68 | 27.8 | ASA III/IV: 258/12 | 95 | Induction: 30 mg.kg-1.h-1 (0-3 min), 10 mg.kg-1.h-1 (3-10 min), 8 mg.kg-1.h-1 (10-20 min), 6 mg.kg-1.h-1 (20 min onwards); Maintenance: 4-10 mg.kg-1.h-1 (from 15 min after skin incision until end of surgery) | 68 | 27 | ASA III/IV: 89/6 |
| Pan 2023[7] | China | operating room | Rigid bronchoscopy: endotracheal tumor resection | Yes(county/nation) | 15 | Induction 0.4 mg/kg, Maintenance 1 mg/kg/h | 61.13 | 20.08 | ASA II/III/IV: 7/7/1 | 15 | Induction 1.5 mg/kg, Maintenance 4-8 mg/kg/h | 60.13 | 21.73 | ASA II/III/IV: 11/4/0 |
| Jeon 2023[8] | South Korea | operating room | Laparoscopic cholecystectomy | Yes(pharmaceutics: Hana Pharmaceuticals, Seoul, South Korea) | 60 | Induction 6 mg/kg/h for LOC; Maintenance 1-2 mg/kg/h to maintain BIS 50 | 70.9 |  | ASA I/II/III: 1/36/23 | 62 | Induction TCI effect-site concentration 4.0 µg/mL; Maintenance 2.5-4.0 µg/mL to maintain BIS 50 | 71.5 |  | ASA I/II/III: 1/29/32 |
| Ju 2024[9] | South Korea | operating room | Coronary artery bypass grafting (CABG) | Yes(pharmaceutics: Hana Pharmaceuticals, Seoul, South Korea) | 50 | Induction: 6 mg/kg/h infusion; Maintenance: 1-2 mg/kg/h infusion | 67 | 25.5 |  | 50 | Induction: 1.5 mg/kg bolus; Maintenance: <1 MAC sevoflurane inhalation | 70 | 23.8 |  |
| Kim 2023[10] | South Korea | operating room | Oral and maxillofacial surgery | Yes(pharmaceutics: Hana Pharmaceuticals, Seoul, South Korea) | 94 | Induction 12 mg/kg/h, Maintenance 1-2 mg/kg/h | 41.7 | 23.8 | ASA I/II: 55/39 | 95 | Induction and Maintenance TCI 3-5 µg/ml | 43.3 | 23.7 | ASA I/II: 54/41 |
| Kim 2024[11] | South Korea | operating room | Spine surgery | Yes(pharmaceutics: Hana Pharmaceuticals, Seoul, South Korea) | 47 | Induction 6 mg/kg/h, Maintenance 1-2 mg/kg/h | 67.4 |  | ASA I/II/III: 0/43/4 | 47 | Induction TCI 2.0 µg/mL (effect-site), increased by 0.5 µg/mL every 30s until LOC; Maintenance TCI 2-3 µg/mL | 67.2 |  | ASA I/II/III: 0/42/5 |
| Kuang 2023[12] | China | operating room | Pulmonary lobectomy, | Yes(country/nation non-commercial) | 42 | Induction 0.3 mg/kg, Maintenance 0.6-1.2 mg/kg/h | 65.4 | 22.4 | ASA I/II: 14/28 | 42 | Induction 2.0 mg/kg, Maintenance 2-10 mg/kg/h | 65.2 | 22.3 | ASA I/II: 11/31 |
| Lan 2024[13] | China | operating room | Urologic surgery | Yes(country/nation non-commercial) | 73 | Induction: 10 mg/kg/h to reach BIS 40-60; Maintenance: 0.2 to 2 mg/kg/h | 46.3 | 24.7 | ASA I/II/III: 9/64/0 | 73 | Induction: 100 mg/min to reach BIS 40-60; Maintenance: plasma concentration 2.5 to 4 mg/ml | 45.4 | 24 | ASA I/II/III: 11/62/0 |
| Lee_2023[14] | South Korea | operating room | Open thyroidectomy | Yes(pharmaceutical company: Hana Pharmaceutical, Seoul, South Korea) | 28 | Induction 6 mg/kg/h, Maintenance 1-2 mg/kg/h | 45 | 24.3 | ASA I/II: 16/12 | 29 | Induction TCI target 3.0 ng/ml, Maintenance TCI target 2.0-3.0 ng/ml (Marsh model) | 51 | 22.6 | ASA I/II: 17/12 |
| Lee 2024(1)[15] | South Korea | operating room | Interventional neuroradiology | Yes(country/nation non-commercial) | 38 | Induction: 12 mg/kg/h continuous infusion; Maintenance: 1-2 mg/kg/h continuous infusion | 56.5 |  | ASA I/II/III: 18/20/0 | 38 | Induction: TCI 4 μg/mL; Maintenance: TCI adjusted to BIS 40-60 | 54.5 |  | ASA I/II/III: 18/19/1 |
| Lee 2024(2)[16] | South Korea | operating room | Breast cancer surgery | Yes(pharmaceutics: Hana Pharmaceuticals, Seoul, South Korea) | 32 | Induction: 6 mg/kg/h continuous infusion; Maintenance: 1 to 2 mg/kg/h continuous infusion | 54 | 23.7 | ASA I/II: 18/14 | 31 | Induction: TCI effect-site concentration up to 4.0 μg/ml (Marsh model); Maintenance: TCI effect-site concentration 4.0 μg/ml (Modified Marsh model) | 54 | 24.1 | ASA I/II: 16/15 |
| Lee J 2024(3)[17] | South Korea | operating room | Spine surgery | No | 36 | Induction: 6-12 mg/kg/h, Maintenance: 1.0-2.0 mg/kg/h | 54.2 |  | ASA I/II/III | 36 | Induction: TCI 3.0 ng/mL (Schnider model) | 50.3 |  | ASA I/II/III |
| Liu 2021[18] | China | operating room | Cardiac valve replacement | Yes(country/nation non-commercial) | 30 | Induction: total dose 0.3 mg/kg, constant speed pump at 1.8 mg/kg/h | 54.9 |  | ASA III: 30 | 30 | Induction: Target-controlled infusion (TCI) 2.5 µg/ml | 50.6 |  | ASA III: 30 |
| Liu T 2024[19] | China | operating room | Laparoscopic cholecystectomy | Yes(country/nation non-commercial) | 50 | Induction: 0.1-0.2 mg/kg; Maintenance: 0.4-1.2 mg/(kg·h) | 71.62 | 23.109 | ASA I/II/III: 27/18/5 | 50 | Induction: 1-2 mg/kg; Maintenance: 4-10 mg/(kg·h) | 71.4 | 23.953 | ASA I/II/III: 29/15/6 |
| Long 2024[20] | China | operating room | Percutaneous balloon compression (PBC) | Yes(country/nation non-commercial) | 40 | Induction 0.2-0.3 mg/kg, Maintenance 0.5-2.0 mg/kg/h | 67 | 23.2 | ASA I-II: 17, ASA III-IV: 23 | 40 | Induction 2.0-2.5 mg/kg, Maintenance 2-8 mg/kg/h | 69 | 24 | ASA I-II: 21, ASA III-IV: 19 |
| Luo 2023[21] | China | operating room | Laparoscopic cholecystectomy | Yes(country/nation non-commercial) | 143 | Induction 9.0 mg/kg/h, Maintenance 2 mg/kg/h; | |  | ASA I/II | 49 | Induction 2.0 mg/kg, Maintenance 6.0 mg/kg/h | 37.37 | 23.01 | ASA I/II: |
| Mao 2022[22] | China | operating room | Urologic surgery | No | 64 | Induction 0.2-0.3 mg/kg, Maintenance 1-2 mg/kg/h | 52.5 | 25.2 | ASA I/II/III: 11/45/8 | 64 | Induction 2-3 mg/kg, Maintenance 4-10 mg/kg/h | 50 | 23.7 | ASA I/II/III: 19/36/9 |
| Matsumoto 2023[23] | Japan | operating room | Laparoscopic cholecystectomy | No | 30 | Induction 12 mg/kg/h, Maintenance 0.4-1 mg/kg/h | 50 |  | ASA I/II/III: 9/21/0 | 30 | Induction TCI effect-site concentration 3 µg/mL, Maintenance TCI to maintain BIS 40-60 | 46.5 |  | ASA I/II/III: 11/19/0 |
| Oh 2023[24] | South Korea | operating room | Catheter ablation for atrial fibrillation with laryngeal mask airway ventilation. | No | 50 | Induction 6 mg/kg/h, Maintenance 1.0-2.0 mg/kg/h | 60 | 26 | ASA II/III: 38/12 | 50 | Induction TCI 5.0 μg/ml, Maintenance TCI 3.0-5.0 μg/ml | 60 | 24.7 | ASA II/III: 37/13 |
| Kotani 2024[25] | Japan | operating room | Transcatheter aortic valve replacement (TAVR), | No | 17 | Induction 12 mg/kg/h, Maintenance adjusted by PSI (25-50) using SedLine monitor | 82.7 |  |  | 18 | Induction TCI 2.5 mcg/mL (Marsh model), Maintenance adjusted by PSI (25-50) using SedLine monitor | 84.7 |  |  |
| Zhang 2022[26] | China | operating room | Hip replacement surgery | Yes(country/nation non-commercial) | 30 | Induction 0.2-0.4 mg/kg, Maintenance 0.3-0.5 mg/kg/h | 74.31 | 24.07 | ASA II/III: 14/16 | 29 | Induction 1.5-2 mg/kg, Maintenance 4-8 mg/kg/h | 75.04 | 23.99 | ASA II/III: 13/16 |
| Luo 2023[27] | China | operating room | Day-case surgery | Yes(country/nation non-commercial) | 38 | Induction 0.3 mg/kg, Maintenance 1-3 mg/kg/h | 43.5 | 22.7 | ASA I/II: 17/21 | 38 | Induction 2.0-2.5 mg/kg, Maintenance 6-12 mg/kg/h | 44.3 | 23.2 | ASA I/II: 19/19 |
| Huang 2023[28] | China | operating room | Breast cancer surgery | Yes(country/nation non-commercial) | 60 | Induction 0.3 mg/kg, Maintenance 0.3 mg/kg/h | 62.6 | 24.3 | ASA II/III: 32/28 | 60 | Induction 2 mg/kg, Maintenance 2 mg/kg/h | 63.8 | 24.8 | ASA II/III: 24/36 |
| Luo_2024[29] | China | operating room | Laparoscopic cholecystectomy | Yes(county/nation) | 56 | Induction 0.3 mg/kg, Maintenance 1.0-2.0 mg/kg/h | 42.8 | 23.9 | ASA I/II: 45/11 | 56 | Induction 2.0 mg/kg, Maintenance 4-10 mg/kg/h | 43 | 23.9 | ASA I/II: 48/8 |
| Lee2024[30] | South Korea | operating room | Radiofrequency ablation for atrial fibrillation with supraglottic airway or tracheal intubation | Yes(pharmaceutics: Hana Pharmaceuticals, Seoul, South Korea) | 26 | Induction 6 mg/kg/h, Maintenance 1-2 mg/kg/h | 66 | 24.6 |  | 27 | TCI target effect-site concentration 3-4 μg/mL | 58 | 26.1 |  |
| Sato 2024[31] | Japan | operating room | Neurosurgical procedures | No | 26 | Induction: 12 mg/kg/h continuous infusion, reduced after loss of consciousness; Maintenance: 0.5 to 1.0 mg/kg/h | 41 | 22.5 | ASA I/II/III | 26 | Induction: 3 μg/mL target-controlled infusion; Maintenance: 2.5 to 4.5 μg/mL target control infusion | 41.5 | 21.6 | ASA I/II/III: |
| Sekiguchi 2023[32] | Japan | operating room | No surgery type was declared (surgery with intubation) | Yes(country/nation non-commercial) | 20 | Induction 12 mg/kg/h, Maintenance 1-2 mg/kg/h | 67 | 23.7 | ASA I/II: 5/15 | 20 | Induction 3 μg/mL (TCI), Maintenance 2-5 μg/mL | 62 | 23.1 | ASA I/II: 4/16 |
| Shimizu 2023[33] | Japan | operating room | Endoscopic sinus surgery | No | 32 | Induction 12 mg/kg/h, Maintenance 1-2 mg/kg/h | 43.5 | 23.3 | ASA I/II: 19/13 | 32 | Induction TCI 3-4 μg/ml, Maintenance TCI 2-5 μg/ml | 44.4 | 23.2 | ASA I/II: 22/10 |
| So 2023[34] | South Korea | operating room | Laparoscopic cholecystectomy | Yes(country/nation non-commercial) | 42 | Induction 6 mg/kg/h until LOC, Maintenance 1 mg/kg/h (max 2 mg/kg/h) | 74.5 | 24.9 | ASA I/II/III: 4/35/3 | 39 | Induction 1.0-1.5 mg/kg over 1 min, Maintenance 100 μg/kg/min | 76 | 24 | ASA I/II/III: 6/27/6 |
| Takaki 2024[35] | Japan | operating room | No surgery type was declared (surgery with intubation) | No | 43 | Induction 12 mg/kg/h continuous infusion until loss of consciousness | 82 | 23.4 | ASA I/II/III: 41/2 | 44 | Induction 0.025 mg/kg/s continuous infusion until loss of consciousness | 82 | 22.9 | ASA I/II/III: 42/2 |
| Toyota 2023[36] | Japan | operating room | Spine surgery | Yes(country/nation non-commercial) | 20 | Induction: 12 mg/kg/h continuous infusion; Maintenance: adjusted to keep BIS/state entropy 40-60, maximum 2 mg/kg/h | 80 | 23.6 | ASA II/III: 18/2 | 19 | Induction: target-controlled infusion initial target 3 µg/mL; Maintenance: adjusted to keep BIS/state entropy 40-60 | 81 | 22.9 | ASA II/III: 15/4 |
| Xing 2024[37] | China | operating room | Laparoscopic cholecystectomy | No | 31 | Induction 0.2 mg/kg, Maintenance 0.4-1.2 mg/kg/h | 54.8 | 23.5 | ASA I/II: 6/25 | 33 | Induction 2 mg/kg, Maintenance 4-10 mg/kg/h | 53.9 | 24.6 | ASA I/II: 7/26 |
| Xu 2023[38] | China | operating room | Orthopedic surgery | Yes(country/nation non-commercial) | 30 | Induction 0.2 mg/kg, plus 0.05 mg/kg adjusted to BIS 40-60 | 69.9 | 21.5 | ASA II/III | 30 | Induction 1.5 mg/kg, plus 1 mg/kg to adjust BIS to 40-60 | 68.6 | 22.2 | ASA II/III |
| Yang 2023[39] | China | operating room | Orthopedic surgery | Yes(country/nation non-commercial) | 147 | Induction 0.2-0.3 mg/kg, Maintenance intravenous infusion adjusted to BIS 40-60 | 68 | 25 | ASA I/II/III: 4/112/31 | 153 | Induction 1.0-1.5 mg/kg, Maintenance intravenous infusion adjusted to BIS 40-60 | 68 | 25.3 | ASA I/II/III: 8/120/25 |
| Zhang 2024[40] | China | operating room | Day-case surgery | No | 65 | Induction 0.3 mg/kg, Maintenance 1-1.5 mg/kg/h | 49.08 | 24.23 | ASA I/II: 30/35 | 63 | Induction 2-2.5 mg/kg, Maintenance 4-8 mg/kg/h | 47.63 | 23.73 | ASA I/II: 38/25 |
| Zhang 2023[41] | China | operating room | Cerebrovascular interventional surgery | Yes(country/nation non-commercial) | 71 | Induction 0.1 mg/kg, Maintenance 0.3-0.7 mg/kg/h | 56.6 | 24.2 | ASA I/II/III: 1/64/6 | 71 | Induction 1-1.5 mg/kg, Maintenance 4-10 mg/kg/h | 56 | 24.5 | ASA I/II/III: 1/63/7 |
| Tang 2023[42] | China | ICU | Mechanical ventilation, deep sedation in critically ill patients | No | 30 | Induction: null, Maintenance: initial 0.3 mg/kg/h, adjusted (max 3.0 mg/kg/h) | 62 | 23.1 |  | 30 | Induction: null, Maintenance: initial 3.0 mg/kg/h, adjusted (max 12.0 mg/kg/h) | 64.5 | 24.1 |  |

**Table S2. Characteristics of Analysis B included studies (13 RCTs)**

| **study_id** | **country** | **clinical_setting** | **surgery/operation** | **funding_status** | **remi_n** | **remi_dose** | **remi_age (mean/median)** | **remi_bmi (mean/median)** | **remi_asa_status** | **DEX_n** | **DEX_dose** | **DEX_age (mean/median)** | **DEX_bmi (mean/median)** | **DEX_asa_status** |
| --- | --- | --- | --- | --- | --- | --- | --- | --- | --- | --- | --- | --- | --- | --- |
| Chen 2022[43] | China | Endoscope | Flexible bronchoscopy with sedation | null | 73 | Induction: 12 mg/kg/h for 10 min; Maintenance: 1-2 mg/kg/h | 57.05 | 24.58 | ASA I/II: 25/48 | 73 | Induction: 0.5 μg/kg for 10 min; Maintenance: 0.2-0.7 μg/kg/h | 56.05 | 25.11 | ASA I/II: 27/46 |
| Chen 2024[44] | China | Operating room | Lower limb orthopedic surgery with sedation | Yes(country/nation non-commercial) | 40 | Induction: 0.03 mg/kg/min; Maintenance: 0.2-0.5 mg/kg/h | 58.77 | 22.72 | ASA I/II/III: 11/20/9 | 40 | Induction: 0.3 µg/kg; Maintenance: 0.2-1.0 µg/kg/h | 61.7 | 23.18 | ASA I/II/III: 10/19/11 |
| Chen2024[45] | China | Operating room | Oral and maxillofacial surgery with tracheal intubation | Yes(country/nation non-commercial) | 59 | Group R1S: Induction 0.073 mg/kg; Group R2S: Induction 0.093 mg/kg |  |  | ASA I/II/III: 13/44/2 | 30 | Induction 0.6 µg/kg | 48.97 | 21.63 | ASA I/II/III: 3/25/2 |
| Deng 2022[46] | China | ICU | Orthopedic surgery (hip arthroplasty, internal fixation); all patients underwent surgery under general anesthesia with laryngeal mask or endotracheal intubation | Yes(country/nation non-commercial) | 37 | Loading dose 0.075 mg/kg over 1 minute, followed by continuous infusion 0.1 to 0.3 mg/kg/h (RASS-guided titration) | 81.5 |  |  | 38 | Loading infusion 0.5 μg/kg over 10 minutes, followed by maintenance dose 0.2 to 0.7 μg/kg/h (RASS-guided titration) | 82.3 |  |  |
| Deng 2023[47] | China | Operating room | Knee arthroplasty/ hip arthroplasty with sedation | Yes(country/nation non-commercial) | 54 | Loading dose 0.025-0.1 mg/kg, Maintenance 0.1-1.0 mg/kg/h | 70.8 | 26.3 | ASA II/III: 31/23 | 54 | 0.2-0.7 μg/kg/h | 71.8 | 26.2 | ASA II/III: 35/19 |
| Hong 2024[48] | Korea | Operating room | Lower limb orthopedic surgery with sedation | Yes(country/nation non-commercial) | 35 | Induction: 6 mg/kg/h for 10 min, Maintenance: 1 mg/kg/h | 39 |  |  | 35 | Induction: 6 µg/kg/h for 10 min, Maintenance: 1 µg/kg/h | 38 |  |  |
| Kim H 2024[49] | South Korea | Operating room | Lower limb orthopedic surgery with sedation | Yes(pharmaceutics: Hana Pharm) | 52 | Induction 0.075 mg/kg over 1 min, Maintenance 0.5 mg/kg/h (range 0.5~1.0 mg/kg/h) | 52 | 24.9 | ASA I/II: 15/37 | 51 | Induction 1 µg/kg over 10 min, Maintenance 0.5 µg/kg/h (range 0.2–0.7 µg/kg/h) | 54 | 26.1 | ASA I/II: 11/40 |
| Lee 2023[50] | Republic of Korea | Operating room | Upper or lower limb surgery with sedation | No | 39 | Induction: 2.5 mg IV over 1 min, additional 2.5 mg if needed; Maintenance: 0.1-1 mg/kg/h continuous IV infusion | 60 |  | ASA I/II/III: 9/20/10 | 39 | Induction: 1 µg/kg IV over 10 min; Maintenance: 0.2-0.7 µg/kg/h continuous IV infusion | 58 | 23.9 | ASA I/II/III: 10/16/13 |
| Li_2024[51] | China | Operating room | Endoscopic sinus surgery with tracheal intubation | No | 40 | Loading 0.075 mg/kg, Maintenance 0.1 mg/kg/h | 40.7 |  | ASA I/II: 25/15 | 40 | Loading 1.0 µg/kg, Maintenance 0.5 µg/kg/h | 42.4 |  | ASA I/II: 28/12 |
| Liao 2023[52] | China | Operating room | Laparoscopic radical gastrectomy for gastric cancer | Yes(National Natural Science Foundation of China, Beijing, China) | 34 | Induction: remimazolam mesylate 0.2 mg/kg; Maintenance: remimazolam 0.3-0.5 mg/kg/h | 70.12 | 21.74 | ASA II/III | 35 | Induction: dexmedetomidine 200 μg diluted to 50 mL with saline (4 μg/mL), pumped 0.5 μg/kg over 10 min before induction; Maintenance: dexmedetomidine 0.3-0.5 μg/kg/h | 71.26 | 21.46 | ASA II/III |
| Xiao 2022[53] | China | Operating room | Abdominal tumor surgery, ultrasound-guided nerve block with sedation | Yes(country/nation non-commercial) | 50 | Induction 5 mg, Rescue 2.5 mg | 56.1 | 22.4 | ASA I/II/III | 50 | Induction 0.6 µg/kg, Rescue 0.4 µg/kg | 57.9 | 22.1 | ASA I/II/III |
| Xu2024[54] | China | Endoscope | Flexible bronchoscopy with sedation | Yes(country/nation non-commercial) | 60 | Induction 6 mg/kg/h, Maintenance 1-2 mg/kg/h | 60.7 | 24.1 | ASA I/II: 22/38 | 60 | Induction 0.5 μg/kg for 10 min, Maintenance 0.2-0.7 μg/kg/h | 60.9 | 24.5 | ASA I/II: 23/37 |
| Zhou2024[55] | China | Endoscope | Flexible bronchoscopy with sedation | Yes(country/nation non-commercial) | 182 | 0.1-0.2 mL/kg infution 3-10 min; maintenance：0.1-0.5 mL/kg/h | 60 | 22.6 | ASA I/II/III: 6/162/14 | 181 | 0.1-0.2 mL/kg infusion 5-10min; maintenance：0.1-0.5 mL/kg/h | 61 | 22.24 | ASA I/II/III: 10/164/8 |

References

1. Choi J, Lee H, Kim J, Han D, Yang J, Kim M, et al. Comparison of remimazolam-based and propofol-based total intravenous anesthesia on postoperative quality of recovery: A randomized non-inferiority trial. JOURNAL OF CLINICAL ANESTHESIA. 2022;82. https://doi.org/10.1016/j.jclinane.2022.110955.

2. Choi SH, Min KT, Park EK, Park S. Comparison of hypotension incidence between remimazolam and propofol in patients with hypertension undergoing neurosurgery: prospective, randomized, single-blind trial. BMC Anesthesiology. 2024;24:198. https://doi.org/10.1186/s12871-024-02578-7.

3. Doi M, Morita K, Takeda J, Sakamoto A, Yamakage M, Suzuki T. Efficacy and safety of remimazolam versus propofol for general anesthesia: a multicenter, single-blind, randomized, parallel-group, phase IIb/III trial. J Anesth. 2020;34:543–53. https://doi.org/10.1007/s00540-020-02788-6.

4. Duan J, Ju X, Wang X, Liu N, Xu S, Wang S. Effects of Remimazolam and Propofol on Emergence Agitation in Elderly Patients Undergoing Hip Replacement: A Clinical, Randomized, Controlled Study. Drug Des Devel Ther. 2023;17:2669–78. https://doi.org/10.2147/DDDT.S419146.

5. Fang P-P, Hu J, Wei Q-F, Liang Y-J, Fan Y-G, Shen Q-Y, et al. Effect of remimazolam besylate vs propofol on incidence of postoperative delirium in older patients undergoing hip surgery: a randomized Non-inferiority trial. Int J Surg. 2024. https://doi.org/10.1097/JS9.0000000000001908.

6. Fechner J, El-Boghdadly K, Spahn DR, Motsch J, Struys MMRF, Duranteau O, et al. Anaesthetic efficacy and postinduction hypotension with remimazolam compared with propofol: a multicentre randomised controlled trial. Anaesthesia. 2024;79:410–22. https://doi.org/10.1111/anae.16205.

7. Pan Y, Chen M, Gu F, Chen J, Zhang W, Huang Z, et al. Comparison of Remimazolam-Flumazenil versus Propofol for Rigid Bronchoscopy: A Prospective Randomized Controlled Trial. Journal of Clinical Medicine. 2022;12:257. https://doi.org/10.3390/jcm12010257.

8. Jeon Y-G, Kim S, Park J-H, Lee J, Song SA, Lim HK, et al. Incidence of intraoperative hypotension in older patients undergoing total intravenous anesthesia by remimazolam versus propofol: A randomized controlled trial. Medicine (Baltimore). 2023;102:e36440. https://doi.org/10.1097/MD.0000000000036440.

9. Ju J-W, Lee DJ, Chung J, Lee S, Cho YJ, Jeon Y, et al. Effect of remimazolam versus propofol on hypotension after anesthetic induction in patients undergoing coronary artery bypass grafting: A randomized controlled trial. Journal of Clinical Anesthesia. 2024;98:111580. https://doi.org/10.1016/j.jclinane.2024.111580.

10. Kim E-J, Kim C-H, Yoon J-Y, Byeon G-J, Kim HY, Choi E-J. Comparison of postoperative nausea and vomiting between Remimazolam and Propofol in Patients undergoing oral and maxillofacial surgery: a prospective Randomized Controlled Trial. BMC Anesthesiology. 2023;23:132. https://doi.org/10.1186/s12871-023-02091-3.

11. Kim H-J, Kim J-Y, Park H-S, Kim H, Ro Y-J, Koh WU. Effect of Remimazolam- versus Propofol-Based Total Intravenous General Anesthesia on Intraoperative Hemodynamic Stability for Major Spine Surgery in the Prone Position: A Randomized Controlled Trial. Medicina (Kaunas). 2024;60:432. https://doi.org/10.3390/medicina60030432.

12. Kuang Q, Zhong N, Ye C, Zhu X, Wei F. Propofol Versus Remimazolam on Cognitive Function, Hemodynamics, and Oxygenation During One-Lung Ventilation in Older Patients Undergoing Pulmonary Lobectomy: A Randomized Controlled Trial. J Cardiothorac Vasc Anesth. 2023;37:1996–2005. https://doi.org/10.1053/j.jvca.2023.06.027.

13. Lan H, Cao H, Liu S, Gong X, Huang X, Rong H, et al. Efficacy of remimazolam tosilate versus propofol for total intravenous anaesthesia in urological surgery: A randomised clinical trial. European Journal of Anaesthesiology | EJA. 2024;41:208. https://doi.org/10.1097/EJA.0000000000001938.

14. Lee H-J, Lee HB, Kim YJ, Cho H-Y, Kim WH, Seo J-H. Comparison of the recovery profile of remimazolam with flumazenil and propofol anesthesia for open thyroidectomy. BMC Anesthesiol. 2023;23:147. https://doi.org/10.1186/s12871-023-02104-1.

15. Lee JH, Lee J, Park SH, Han S-H, Kim J-H, Park J-W. Comparison between remimazolam and propofol anaesthesia for interventional neuroradiology: a randomised controlled trial. Anaesthesia Critical Care & Pain Medicine. 2024;43:101337. https://doi.org/10.1016/j.accpm.2023.101337.

16. Lee J, Kim D, Ju J, Nam K, Cho Y, Jeon Y, et al. Comparison of recovery profiles between total intravenous anaesthesia with propofol or remimazolam reversed with flumazenil in patients undergoing breast surgery A randomised controlled trial. EUROPEAN JOURNAL OF ANAESTHESIOLOGY. 2024;41:199–207. https://doi.org/10.1097/EJA.0000000000001951.

17. Lee J, Han DW, Song Y, Lee J, Jeon S, Kim MH. Quality of postoperative recovery in total intravenous anesthesia between remimazolam and propofol for intraoperative neurophysiological monitoring: A prospective double-blind randomized controlled trial. J Pers Med. 2024;14. https://doi.org/10.3390/jpm14040382.

18. Liu T, Lai T, Chen J, Lu Y, He F, Chen Y, et al. Effect of remimazolam induction on hemodynamics in patients undergoing valve replacement surgery: A randomized, double-blind, controlled trial. PHARMACOLOGY RESEARCH & PERSPECTIVES. 2021;9. https://doi.org/10.1002/prp2.851.

19. Liu T, Zhao H, Zhao X, Qu M. Comparison of Remimazolam and Propofol on Postoperative Delirium in Elderly Patients Undergoing Radical Resection of Colon Cancer: A Single-Center Prospective Randomized Controlled Study. Med Sci Monit. 2024;30:e943784. https://doi.org/10.12659/MSM.943784.

20. Long D, Chen K, Li Y, He P, Li X, Qin X, et al. Comparison of Remimazolam and Propofol for Intravenous Anesthesia on Trigeminocardiac Reflex in Percutaneous Balloon Compression for Trigeminal Neuralgia: A Randomized Controlled Trial. DRUG DESIGN DEVELOPMENT AND THERAPY. 2024;18:5225–37. https://doi.org/10.2147/DDDT.S473700.

21. Luo L, Jiang J, Zhang M, Guo Z, Zhang X, Wang F, et al. Comparative Study About Different Doses of Remimazolam in Short Laparoscopic Surgery: A Randomized Controlled Double-Blind Trial. THERAPEUTICS AND CLINICAL RISK MANAGEMENT. 2023;19:829–37. https://doi.org/10.2147/TCRM.S428278.

22. Mao YY, Guo J, Yuan JJ, Zhao EX, Yang JJ. Quality of Recovery After General Anesthesia with Remimazolam in Patients’ Undergoing Urologic Surgery: A Randomized Controlled Trial Comparing Remimazolam with Propofol. DRUG DESIGN DEVELOPMENT AND THERAPY. 2022;16:1199–209. https://doi.org/10.2147/DDDT.S359496.

23. Matsumoto A, Satomi S, Kakuta N, Narasaki S, Toyota Y, Miyoshi H, et al. Remimazolam’s Effects on Postoperative Nausea and Vomiting Are Similar to Those of Propofol after Laparoscopic Gynecological Surgery: A Randomized Controlled Trial. JOURNAL OF CLINICAL MEDICINE. 2023;12. https://doi.org/10.3390/jcm12165402.

24. Oh EJ, Chung YJ, Lee J-H, Kwon EJ, Choi EA, On YK, et al. Comparison of propofol vs. remimazolam on emergence profiles after general anesthesia: A randomized clinical trial. Journal of Clinical Anesthesia. 2023;90:111223. https://doi.org/10.1016/j.jclinane.2023.111223.

25. Kotani T, Ida M, Naito Y, Kawaguchi M. Comparison of remimazolam-based and propofol-based total intravenous anesthesia on hemodynamics during anesthesia induction in patients undergoing transcatheter aortic valve replacement: a randomized controlled trial. Journal of Anesthesia. 2024;38:330–8. https://doi.org/10.1007/s00540-024-03311-x.

26. Zhang J, Wang X, Zhang Q, Wang Z, Zhu S. Application effects of remimazolam and propofol on elderly patients undergoing hip replacement. BMC Anesthesiol. 2022;22:118. https://doi.org/10.1186/s12871-022-01641-5.

27. Luo W, Sun M, Wan J, Zhang Z, Huang J, Zhang J, et al. Efficacy and safety of remimazolam tosilate versus propofol in patients undergoing day surgery: a prospective randomized controlled trial. BMC Anesthesiol. 2023;23:182. https://doi.org/10.1186/s12871-023-02092-2.

28. Huang Y, Yan T, Lu G, Luo H, Lai Z, Zhang L. Efficacy and safety of remimazolam compared with propofol in hypertensive patients undergoing breast cancer surgery: a single-center, randomized, controlled study. BMC Anesthesiology. 2023;23. https://doi.org/10.1186/s12871-023-02364-x.

29. Luo Z, Cao H, Luo L, Chen L, Feng D, Huang G. Comparison of remimazolam tosilate and propofol during induction and maintenance of general anesthesia in patients undergoing laparoscopic cholecystectomy: a prospective, single center, randomized controlled trial. BMC Anesthesiology. 2024;24. https://doi.org/10.1186/s12871-024-02614-6.

30. Lee S, Lee J, Hwang SY, Ju JW, Nam K, Ahn HJ, et al. Remimazolam-flumazenil provides fast recovery from general anesthesia compared to propofol during radiofrequency catheter ablation of atrial fibrillation. Scientific Reports. 2024;14. https://doi.org/10.1038/s41598-024-63578-8.

31. Sato T, Ando T, Ozeki K, Asano I, Kuwatsuka Y, Ando M, et al. Prospective Randomized Controlled Trial Comparing Anesthetic Management With Remimazolam Besylate and Flumazenil Versus Propofol During Awake Craniotomy Following an Asleep-awake-asleep Method. Journal of Neurosurgical Anesthesiology. 2025;37:40. https://doi.org/10.1097/ANA.0000000000000975.

32. Sekiguchi R, Kinoshita M, Kawanishi R, Kakuta N, Sakai Y, Tanaka K. Comparison of hemodynamics during induction of general anesthesia with remimazolam and target-controlled propofol in middle-aged and elderly patients: a single-center, randomized, controlled trial. BMC Anesthesiology. 2023;23:14. https://doi.org/10.1186/s12871-023-01974-9.

33. Shimizu T, Takasusuki T, Yamaguchi S. Remimazolam Compared to Propofol for Total Intravenous Anesthesia with Remifentanil on the Recovery of Psychomotor Function: A Randomized Controlled Trial. ADVANCES IN THERAPY. 2023;40:4395–404. https://doi.org/10.1007/s12325-023-02615-w.

34. So K, Park J, Kim S. Safety and efficacy of remimazolam for general anesthesia in elderly patients undergoing laparoscopic cholecystectomy: a randomized controlled trial. FRONTIERS IN MEDICINE. 2023;10. https://doi.org/10.3389/fmed.2023.1265860.

35. Takaki R, Yokose M, Mihara T, Saigusa Y, Tanaka H, Yamamoto N, et al. Hypotension after general anaesthesia induction using remimazolam or propofol in geriatric patients undergoing sevoflurane anaesthesia with remifentanil: a single-centre, double-blind, randomised controlled trial. British Journal of Anaesthesia. 2024;133:24–32. https://doi.org/10.1016/j.bja.2024.04.013.

36. Toyota Y, Kondo T, Oshita K, Haraki T, Narasaki S, Kido K, et al. Remimazolam-based anesthesia with flumazenil allows faster emergence than propofol-based anesthesia in older patients undergoing spinal surgery: A randomized controlled trial. MEDICINE. 2023;102. https://doi.org/10.1097/MD.0000000000036081.

37. Xing Q, Zhou X, Zhou Y, Shi C, Jin W. Comparison of the effects of remimazolam tosylate and propofol on immune function and hemodynamics in patients undergoing laparoscopic partial hepatectomy: a randomized controlled trial. BMC Anesthesiology. 2024;24:205. https://doi.org/10.1186/s12871-024-02589-4.

38. Xu Q, Wu J, Shan W, Duan G, Lan H. Effects of remimazolam combined with sufentanil on hemodynamics during anesthetic induction in elderly patients with mild hypertension undergoing orthopedic surgery of the lower limbs: a randomized controlled trial. BMC Anesthesiology. 2023;23:311. https://doi.org/10.1186/s12871-023-02249-z.

39. Yang J-J, Lei L, Qiu D, Chen S, Xing L-K, Zhao J-W, et al. Effect of Remimazolam on Postoperative Delirium in Older Adult Patients Undergoing Orthopedic Surgery: A Prospective Randomized Controlled Clinical Trial. Drug Des Devel Ther. 2023;17:143–53. https://doi.org/10.2147/DDDT.S392569.

40. Zhang L, Wang Z, Liu Y, Zhang X, Wu Y. Comparison of Remimazolam Tosilate and Propofol Sedation on the Early Postoperative Quality of Recovery in Patients Undergoing Day Surgery: A Prospective Randomized Controlled Trial. Drug Des Devel Ther. 2024;18:1743–54. https://doi.org/10.2147/DDDT.S456675.

41. Zhang J, Zhang J, Wang Y, Bai X, Guo Q, Liu W, et al. Effect of remimazolam vs propofol on emergence from general anesthesia in patients undergoing cerebral endovascular procedures: A randomized controlled, non-inferiority trial. J Clin Anesth. 2024;93:111356. https://doi.org/10.1016/j.jclinane.2023.111356.

42. Tang Y, Gao X, Xu J, Ren L, Qi H, Li R, et al. Remimazolam besylate versus propofol for deep sedation in critically ill patients: a randomized pilot study. CRITICAL CARE. 2023;27. https://doi.org/10.1186/s13054-023-04760-8.

43. Chen X, Xin D, Xu G, Zhao J, Lv Q. The Efficacy and Safety of Remimazolam Tosilate Versus Dexmedetomidine in Outpatients Undergoing Flexible Bronchoscopy: A Prospective, Randomized, Blind, Non-Inferiority Trial. Front Pharmacol. 2022;13 (Chen X.; Xu G.; Zhao J.; Lv Q., 13869530842@163.com) Department of Anaesthesiology, Liaocheng People’s Hospital, Liaocheng, China. https://doi.org/10.3389/fphar.2022.902065.

44. Chen Y, Cai Y, Yu G, Zhang X, Hu T, Xue R. Safety and effcacy of remimazolam tosilate for sedation during combined spinal-epidural anesthesia for orthopedic procedures: a randomized controlled trial. BMC Anesthesiol. 2024;24:75. https://doi.org/10.1186/s12871-024-02451-7.

45. Chen Q, Qin B, Zhang M, Zhou Y, Shi X, Xie Y. The Safety and Efficacy of Remimazolam Compared to Dexmedetomidine for Awake Tracheal Intubation by Flexible Bronchoscopy: A Randomized, Double-Blind, Controlled Trial. Drug Des Devel Ther. 2024;18 (Chen Q.; Xie Y., xybdoctor@163.com) Department of Anesthesiology, The First Affiliated Hospital of Guangxi Medical University, Nanning, China:967–78. https://doi.org/10.2147/DDDT.S446222.

46. Deng Y, Qin Z, Wu Q, Liu L, Yang X, Ju X, et al. Efficacy and Safety of Remimazolam Besylate versus Dexmedetomidine for Sedation in Non-Intubated Older Patients with Agitated Delirium After Orthopedic Surgery: A Randomized Controlled Trial. Drug Des Devel Ther. 2022;16 (Deng Y.; Qin Z., qin18716111836@126.com; Liu L.; Yang X.; Ju X.; Zhang Y.) Intensive Care Unit, Sichuan Provincial Orthopedic Hospital, Chengdu, China:2439–51. https://doi.org/10.2147/DDDT.S373772.

47. Deng C-M, Meng Z-T, Yang J, Zhang C-J, Lu M, Wang Y-X, et al. Effect of intraoperative remimazolam on postoperative sleep quality in elderly patients after total joint arthroplasty: a randomized control trial. J Anesth. 2023;37:511–21. https://doi.org/10.1007/s00540-023-03193-5.

48. Hong S-W, Park J-Y, Rhee K-Y, Kim S-H. Comparison emergence of sedation, using dexmedetomidine and remimazolam, in spinal anaesthesia - double blinded randomized controlled trial. Int J Med Sci. 2024;21:1552–8. https://doi.org/10.7150/ijms.95736.

49. Kim H, Kim Y, Bae J, Yoo S, Lim Y-J, Kim J-T. Comparison of remimazolam and dexmedetomidine for intraoperative sedation in patients undergoing lower extremity surgery under spinal anesthesia: a randomized clinical trial. Reg Anesth Pain Med. 2024;49:110–6. https://doi.org/10.1136/rapm-2023-104415.

50. Lee S, Kim M, Kang HY, Choi J-H, Kim MK, You AH. Comparison of oxygen reserve index according to the remimazolam or dexmedetomidine for intraoperative sedation under regional anesthesia—A single-blind randomized controlled trial. Front Med. 2023;10 (Lee S.; Kim M.; Kang H.Y.; Choi J.-H.; Kim M.K.; You A.H., ahyou@khu.ac.kr) Department of Anesthesiology and Pain Medicine, Kyung Hee University College of Medicine, Kyung Hee University Hospital, Seoul, South Korea. https://doi.org/10.3389/fmed.2023.1288243.

51. Li Y, Zhou H, Gao F, Guan Q, Wang S, Tan Y, et al. Comparison of the effects of remimazolam and dexmedetomidine on the quality of recovery in functional endoscopic sinus surgery: a randomized clinical trial. BMC Anesthesiol. 2024;24:472. https://doi.org/10.1186/s12871-024-02860-8.

52. Liao YQ, Min J, Wu ZX, Hu Z. Comparison of the effects of remimazolam and dexmedetomidine on early postoperative cognitive function in elderly patients with gastric cancer. Front Aging Neurosci. 2023;15 (Liao Y.Q.; Min J., candymin66@163.com; Wu Z.X.; Hu Z.) Department of Anesthesiology, Medical Center of Anesthesiology and Pain, The First Affiliated Hospital of Nanchang University, Jiangxi, Nanchang, China. https://doi.org/10.3389/fnagi.2023.1123089.

53. Xiao Y, Wei R, Chen L, Chen Y, Kong L. Efficacy and safety of remimazolam for procedural sedation during ultrasound-guided transversus abdominis plane block and rectus sheath block in patients undergoing abdominal tumor surgery: a single-center randomized controlled trial. BMC Anesthesiol. 2022;22. https://doi.org/10.1186/s12871-022-01927-8.

54. Xu H, Wang L, Zhu W, Ren C, Liu G, Liu Y. Comparison of the Safety and Efficacy of Remimazolam Besylate versus Dexmedetomidine for Patients Undergoing Fiberoptic Bronchoscopy: A Prospective, Randomized Controlled Trial. Drug Des Devel Ther. 2024;18 (Xu H.; Zhu W.; Ren C.; Liu G.; Liu Y., lycxyl9@sina.com) Department of Anaesthesiology, Liaocheng People’s Hospital, Liaocheng, China:2317–27. https://doi.org/10.2147/DDDT.S460949.

55. Zhou L, Zou J, Li X, Zuo X, Gu M, Sun K, et al. Efficacy and safety of remimazolam versus dexmedetomidine for patients undergoing flexible fiberoptic bronchoscopy: A randomized, clinical trial. J Clin Anesth. 2024;99 (Zhou L.; Zou J.; Li X.; Zuo X.; Gu M.; Sun K.; Fan W.; Yao Y., yuanyuan58@126.com; Yan M., zryanmin@zju.edu.cn) Department of Anesthesiology, The Second Affiliated Hospital, Zhejiang University, Zhejiang, Hangzhou, China:111677. https://doi.org/10.1016/j.jclinane.2024.111677.
