# Supplementary material for: Multidimensional clinical evaluation of remimazolam versus propofol and dexmedetomidine: two systematic reviews and meta-analyses based on differentiated endpoints
Source: BMC Anesthesiol. 2026 May 28;26:447. doi: 10.1186/s12871-026-03951-4 (PMC13397694; doi:10.1186/s12871-026-03951-4)
Supplement: Supplementary file 1 — Supplementary Material 1: Appendix 1: Search strategies for Analysis A&B. Appendix 2: All tables (including extracted datasets, characteristics of studies with references). Appendix 3: All supplementary figures (Fig S1-S8). Appendix 4: PRISMA checklist. [file 12871_2026_3951_MOESM1_ESM.zip › Appendix-2/Table_S5.docx]

**Table S5. Summary of Findings (GRADE) for secondary outcomes of Analysis A & B**

| **Outcomes** | **Studies, Patients** | **OR/MD (95% CI)** | **Certainty of evidence^#^** | **Reasons** |
| --- | --- | --- | --- | --- |
| **Analysis A: Remimazolam vs. Propofol** | | | | |
| Hypotension incidence | 21, 2399 | 0.35; 95% CI (0.29, 0.43) | ⊕⊕⊕○  Moderate | Downgraded one level for serious indirectness (diverse criteria across RCTs) |
| postoperative nausea and vomiting (PONV) | 22, 2632 | 1.14; 95% CI (0.86, 1.51) | ⊕⊕⊕⊕  High | None |
| Extubation time(minute) | 24, 2789 | -0.88; 95% CI (-2.44, 0.69) | ⊕○○○  Very Low | Downgraded three levels: two for very serious inconsistency (I^2^= 99.5%); one for serious indirectness (variability in surgical procedures) |
| **Analysis B: Remimazolam vs. Dexmedetomidine** | | | | |
| Time to full alertnes (min) | 7, 962 | -7.14, 95%CI (-14.70, 0.43) | ⊕○○○  Very Low | Downgraded three levels: two for very serious inconsistency (I^2^= 99.9%); one for serious publication bias. |
| Postoperative Nausea and Vomiting (PONV) | 8, 960 | 1; 95% CI: (0.6, 1.65) | ⊕⊕⊕⊕  High | None |
| Hypotension incidence | 12, 1381 | 0.64; 95% CI: (0.32, 1.30) | ⊕○○○  Very Low | Downgraded three levels: two for very serious inconsistency (I^2^= 75.4%); one for serious indirectness (definition of hypotension differs among RCTs) |
| Respiratory Complication:desaturation | 12,1401 | 0.98; 95% CI: 0(.48, 1.97) | ⊕⊕○○  Low | Downgraded two levels: one for serious inconsistency (I^2^= 63.9%); one for serious indirectness (definition of desaturation differs across RCTs) |
| RIS: The required information size of Trial Sequential Analysis. OR: odds ratio; MD: mean difference; 95%CI, 95% confidence interval. ^#^Certainty of evidence was judged according to GRADE approaches.  Downgrading criteria:   1. If I2>50%, inconsistency is serious, downgrade one level 2. If I2>75%, inconsistency is very serious, downgrade two levels 3. If the definition of specific outcome differs across study, then the indirectness is serious, downgrade one level 4. If TSA showed no reaching RIS, or effect size is wide, then the imprecision is serious, downgrade one level. 5. If publication bias was observed, downgrade one level. | | | | |
