# Supplementary material for: Multidimensional clinical evaluation of remimazolam versus propofol and dexmedetomidine: two systematic reviews and meta-analyses based on differentiated endpoints
Source: BMC Anesthesiol. 2026 May 28;26:447. doi: 10.1186/s12871-026-03951-4 (PMC13397694; doi:10.1186/s12871-026-03951-4)
Supplement: Supplementary file 1 — Supplementary Material 1: Appendix 1: Search strategies for Analysis A&B. Appendix 2: All tables (including extracted datasets, characteristics of studies with references). Appendix 3: All supplementary figures (Fig S1-S8). Appendix 4: PRISMA checklist. [file 12871_2026_3951_MOESM1_ESM.zip › Appendix1.docx]

**1. Search strategies of Analysis A (Remimazolam vs. Propofol)**

**Pubmed**

| No | Search strategy |
| --- | --- |
| 1 | remimazolam[MeSH Terms] OR remimazolam[Title/Abstract] OR "CNS 7056"[Title/Abstract] OR "CNS-7056"[Title/Abstract] |
| 2 | propofol[MeSH Terms] OR propofol[Title/Abstract] OR diprivan[Title/Abstract] |
| 3 | randomized controlled trial[pt] OR controlled clinical trial[pt] OR randomized[tiab] |
| 4 | "Intubation, Intratracheal"[Mesh] OR tracheal intubation[Title/Abstract] OR endotracheal intubation[Title/Abstract] OR "Anesthesia, General"[Mesh] OR general anesthesia[Title/Abstract] OR anesthesia[Title/Abstract] |
| 5 | (protocol OR letter OR comment) [Title/Abstract] |
| 6 | #1 AND #2 AND #3 AND #4 NOT #5 |

Outcome: 108 (to 2024/12/03)

**Web of science**

| No | Search strategy |
| --- | --- |
| 1 | TS=remimazolam OR TS=cns7054 OR TS=bufalo |
| 2 | TS=propofol OR TS=diisoprofol |
| 3 | (TS=tracheal intubation OR TS=endotracheal intubation) OR (TS=general anesthesia OR TS=anesthesia) |
| 4 | (TS="randomized controlled trial" OR (TS=random* AND TS=controlled AND TS=trial) |
| 5 | #1 AND #2 AND #3 AND #4 |

Outcome: 121 (to 2024/12/03)

**Embase**

| No | Search strategy |
| --- | --- |
| 1 | ‘'remimazolam' OR 'remimazolam'/exp OR remimazolam OR 'cns7056'/exp OR cns7056 OR 'byfavo'/exp OR byfavo |
| 2 | 'propofol' OR 'propofol'/exp OR propofol OR 'disoprofol'/exp OR disoprofol |
| 3 | 'tracheal intubation'/exp OR 'tracheal intubation' OR 'endotracheal intubation'/exp OR 'endotracheal intubation' OR 'general anesthesia'/exp OR 'general anesthesia' OR 'anesthesia'/exp OR anesthesia |
| 4 | 'randomized controlled trial'/exp OR 'randomized controlled trial' OR (random* AND controlled AND ('trial'/exp OR trial) |
| 5 | #1 AND #2 AND #3 AND #4 |

Outcome: 295 (to 2024/12/03)

**Cochrane library**

| No | Search strategy |
| --- | --- |
| 1 | remimazolam OR CNS7056 OR byfavo |
| 2 | propofol OR disoprofol |
| 3 | "tracheal intubation" OR "endotracheal intubation" OR ’general anesthesia‘ OR 'surgery' |
| 4 | 'Randomized controlled trial' OR RCT OR randomi* |
| 5 | #1 AND #2 AND #3 AND #4 |

Outcome: 280 (to 2024/12)

**Clinical trials.gov**

Disease: “surgery”

Other terms: "intubation" OR "ventilator" OR "intensive care" OR "ICU" OR "critical care" OR "critically ill" OR ‘general anesthesia’ OR ‘surgery’

Intervention/treatment: "remimazolam" OR "CNS 7056" OR "CNS7056" OR "remimazolam besylate" OR "remimazolam tosilate" OR "propofol"

Outcome: 49 (to 2024/12/03)

**2. Search strategies of Analysis B (Remimazolam vs Dexmedetomidine)**

**Pubmed**

| No | Search strategy |
| --- | --- |
| 1 | remimazolam[MeSH Terms] OR remimazolam[Title/Abstract] OR "CNS 7056"[Title/Abstract] OR "CNS-7056"[Title/Abstract] |
| 2 | dexmedetomidine[Title/Abstract] OR precedex[Title/Abstract] |
| 3 | (sedation[Title/Abstract] OR "Sedation"[Mesh] OR intensive care[Title/Abstract] OR ICU[Title/Abstract]) OR procedural sedation[Title/Abstract] OR ‘perioperative care’[Title/Abstract] OR ‘operating room’[Title/Abstract] OR endoscope OR "Anesthesia, General"[Mesh] OR general anesthesia[Title/Abstract] OR anesthesia[Title/Abstract]) |
| 4 | randomized controlled trial[pt] OR random*[Title/Abstract] AND controlled[Title/Abstract] AND trial[Title/Abstract] |
| 5 | (protocol OR letter OR comment) [Title/Abstract] |
| 6 | #1 AND #2 AND #3 AND #4 NOT #5 |

**Outcome 12(to 2024/12/03)**

**Web of science**

| No | Search strategy |
| --- | --- |
| 1 | TS=remimazolam OR TS=cns7054 OR TS=bufalo |
| 2 | TS= dexmedetomidine l OR TS= precedex |
| 3 | (TS=sedation OR TS="procedural sedation" OR TS=ICU OR TS="intensive care" OR TS= ‘general anesthesia’) |
| 4 | (TS="randomized controlled trial" OR (TS=random* AND TS=controlled AND TS=trial) |
| 5 | #1 AND #2 AND #3 AND #4 |

Outcome: 24 (to 2024/12/03)

**Embase**

| No | Search strategy |
| --- | --- |
| 1 | 'remimazolam' OR 'remimazolam'/exp OR remimazolam OR 'cns7056'/exp OR cns7056 OR 'byfavo'/exp OR byfavo |
| 2 | dexmedetomidine l OR precedex |
| 3 | 'sedation'/exp OR sedation OR 'procedural sedation' OR 'intensive care' OR icu OR 'general anesthesia'/exp OR 'general anesthesia' OR 'anesthesia'/exp OR anesthesia |
| 4 | 'randomized controlled trial'/exp OR 'randomized controlled trial' OR (random* AND controlled AND ('trial'/exp OR trial) |
| 5 | #1 AND #2 AND #3 AND #4 |

Outcome: 40 (to 2024/12/03)

**Cochrane library**

| No | Search strategy |
| --- | --- |
| 1 | remimazolam OR CNS7056 OR byfavo |
| 2 | dexmedetomidine l OR precedex |
| 3 | sedation OR "procedural sedation" OR "intensive care" OR ICU OR "perioperative care" OR 'general anesthesia' |
| 4 | 'Randomized controlled trial' OR RCT OR randomi* |
| 5 | #1 AND #2 AND #3 AND #4 |

Outcome: 11 (to 2024/12)

**Clinical trials.gov**

Disease: (empty)

Other terms: "sedation" OR "procedural sedation" OR "intensive care" OR “perioperative care” OR ‘general anesthesia’

Intervention/treatment: "remimazolam" OR "CNS 7056" OR "CNS7056" OR "remimazolam besylate" OR "remimazolam tosilate" AND "dexmedetomidine"

Outcome: 5 (to 2024/12/03)
