# Supplementary figures and images for: Multidimensional clinical evaluation of remimazolam versus propofol and dexmedetomidine: two systematic reviews and meta-analyses based on differentiated endpoints
Source: BMC Anesthesiol. 2026 May 28;26:447. doi: 10.1186/s12871-026-03951-4 (PMC13397694; doi:10.1186/s12871-026-03951-4)

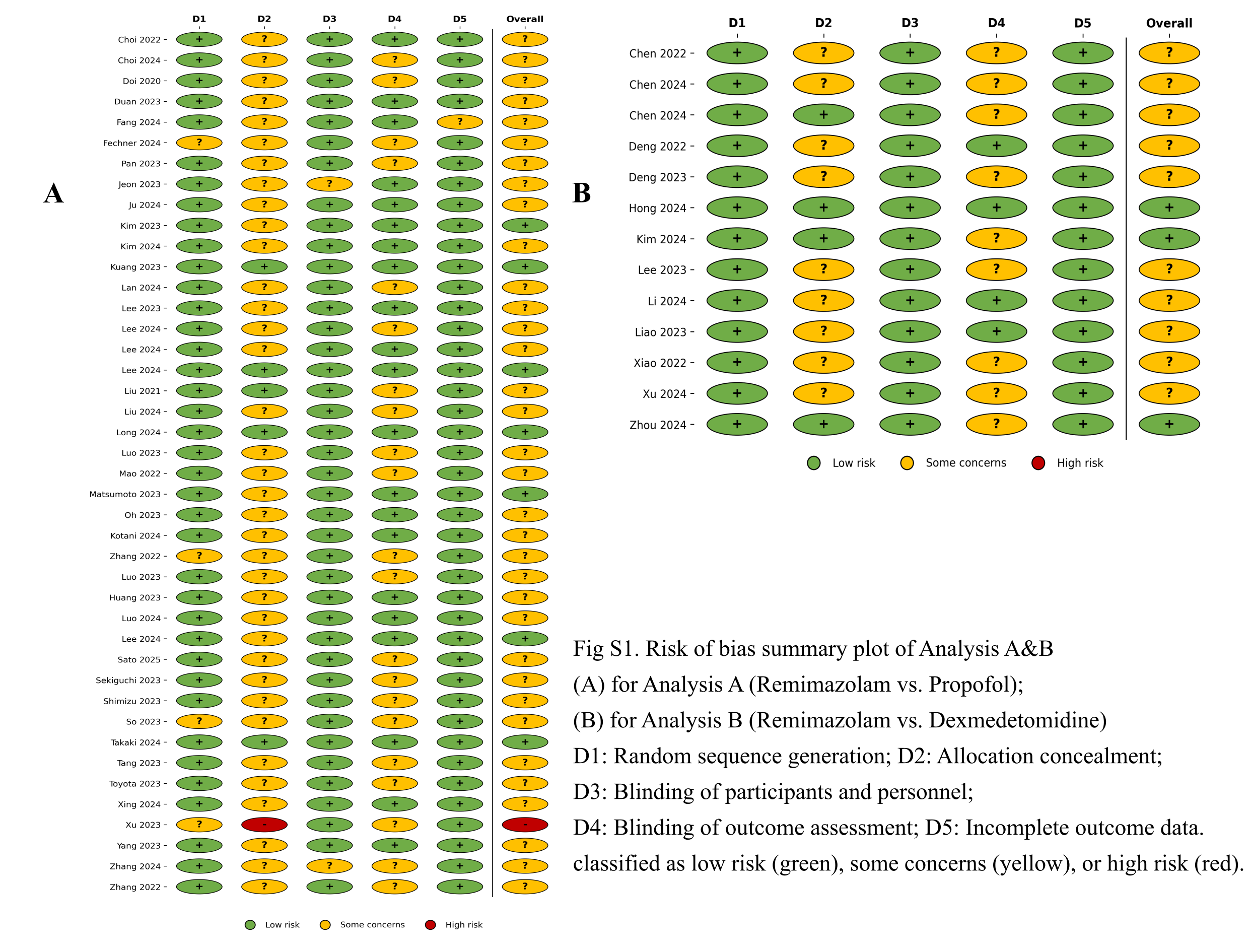

Supplement: Supplementary file 1 — Supplementary Material 1: Appendix 1: Search strategies for Analysis A&B. Appendix 2: All tables (including extracted datasets, characteristics of studies with references). Appendix 3: All supplementary figures (Fig S1-S8). Appendix 4: PRISMA checklist. [file 12871_2026_3951_MOESM1_ESM.zip › Appendix-3/fig_S1.png]

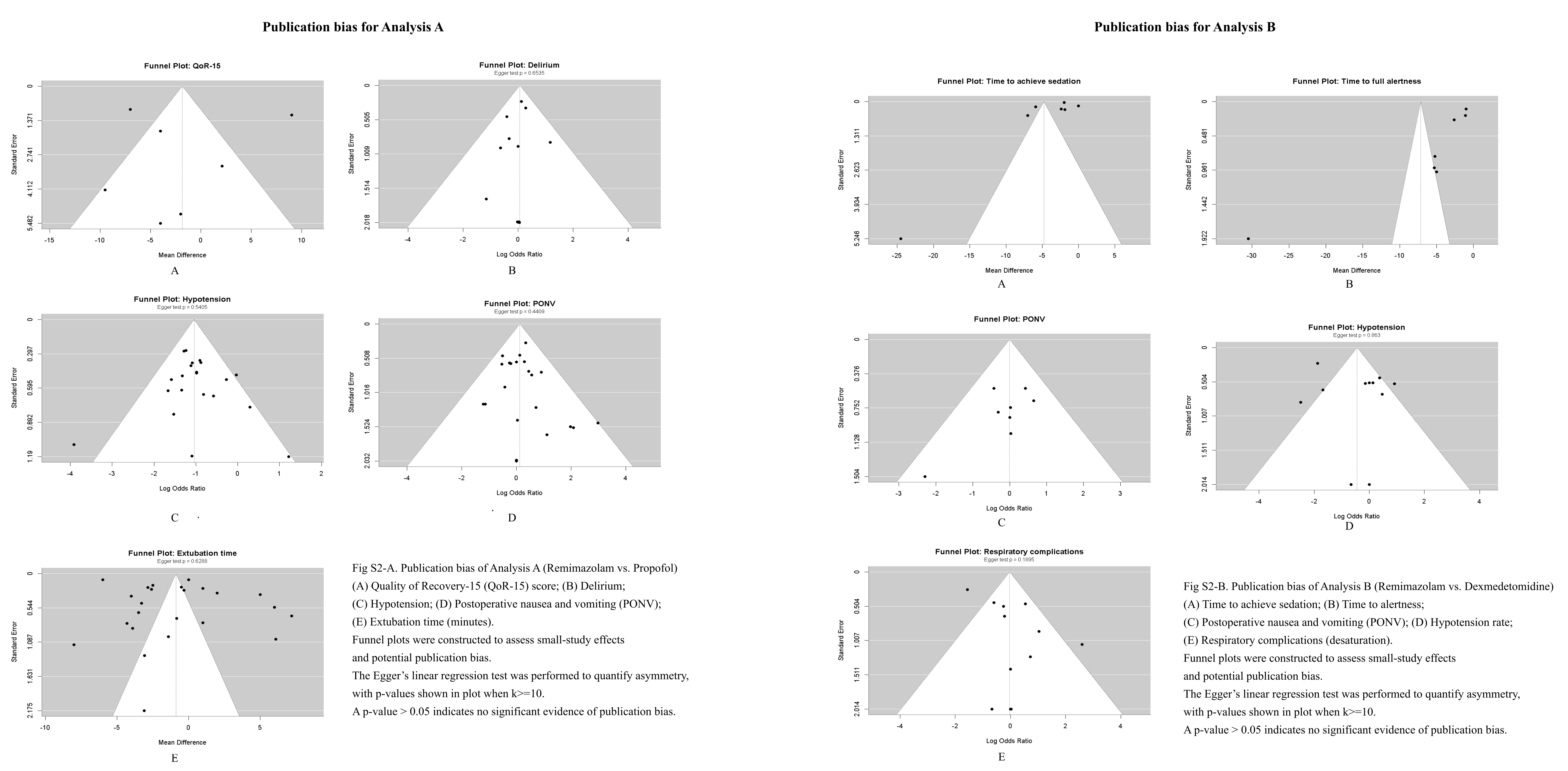

Supplement: Supplementary file 1 — Supplementary Material 1: Appendix 1: Search strategies for Analysis A&B. Appendix 2: All tables (including extracted datasets, characteristics of studies with references). Appendix 3: All supplementary figures (Fig S1-S8). Appendix 4: PRISMA checklist. [file 12871_2026_3951_MOESM1_ESM.zip › Appendix-3/fig_S2.png]

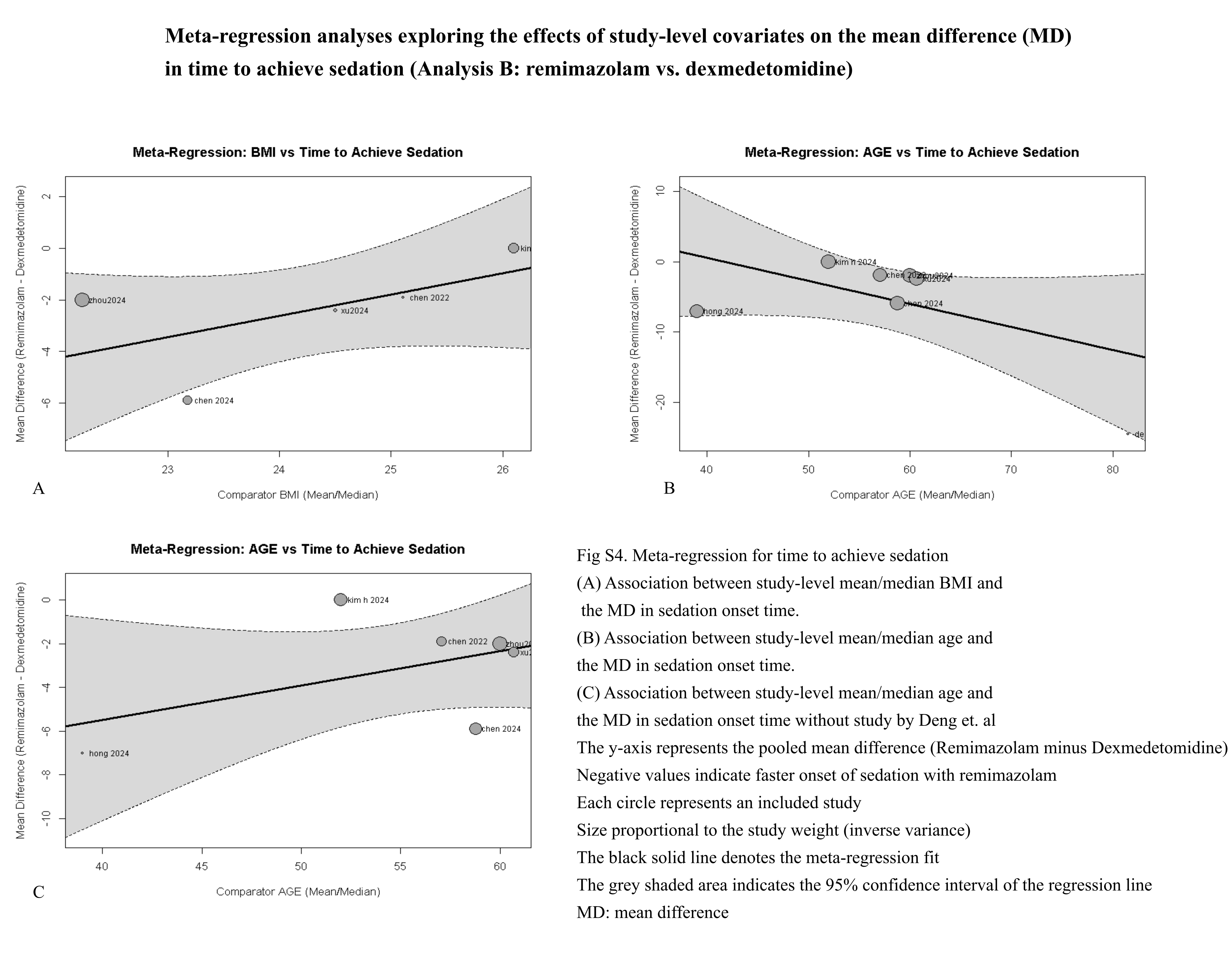

Supplement: Supplementary file 1 — Supplementary Material 1: Appendix 1: Search strategies for Analysis A&B. Appendix 2: All tables (including extracted datasets, characteristics of studies with references). Appendix 3: All supplementary figures (Fig S1-S8). Appendix 4: PRISMA checklist. [file 12871_2026_3951_MOESM1_ESM.zip › Appendix-3/fig_S4.png]

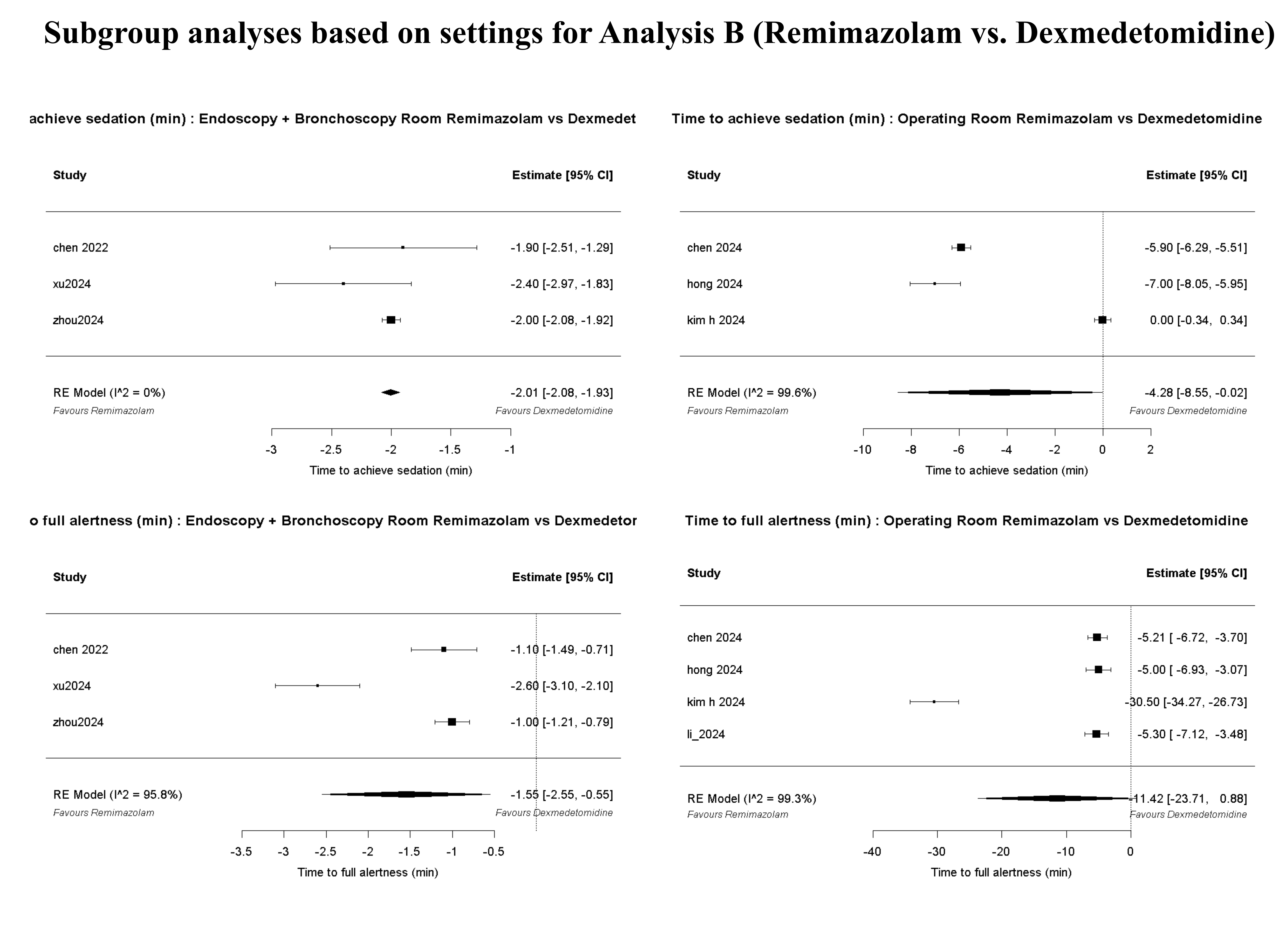

Supplement: Supplementary file 1 — Supplementary Material 1: Appendix 1: Search strategies for Analysis A&B. Appendix 2: All tables (including extracted datasets, characteristics of studies with references). Appendix 3: All supplementary figures (Fig S1-S8). Appendix 4: PRISMA checklist. [file 12871_2026_3951_MOESM1_ESM.zip › Appendix-3/fig_S8.png]
